# Functionalization of Magnetic Chitosan Particles for the Sorption of U(VI), Cu(II) and Zn(II)—Hydrazide Derivative of Glycine-Grafted Chitosan

**DOI:** 10.3390/ma10050539

**Published:** 2017-05-16

**Authors:** Mohammed F. Hamza, Mohsen M. Aly, Adel A.-H. Abdel-Rahman, Samar Ramadan, Heba Raslan, Shengye Wang, Thierry Vincent, Eric Guibal

**Affiliations:** 1 Nuclear Materials Authority, 530 El Maadi, Cairo, Egypt; M_fouda21@hotmail.com (M.F.H.); Prof_mohsennma@yahoo.com (M.M.A.); 2 Ecole des Mines d’Alès, Centre des Matériaux des Mines d’Alès, 6 Avenue de Clavières, F-30319 Ales CEDEX, France; shengye.wang@mines-ales.fr (S.W.); thierry.vincent@mines-ales.fr (T.V.); 3 Faculty of Nursing, Menoufia University, Al Minufya, 00123 Shebine El-Koam, Egypt; adelnassar63@yahoo.com (A.A.-H.A.-R.); samarramadan2007@yahoo.com (S.R.); boba_ras2007@yahoo.com (H.R.)

**Keywords:** magnetic sorbent, chemical modification of chitosan, uranium, copper, zinc, sorption isotherms, uptake kinetics, metal desorption, sorbent recycling, physico-chemical characterization.

## Abstract

A new magnetic functionalized derivative of chitosan is synthesized and characterized for the sorption of metal ions (environmental applications and metal valorization). The chemical modification of the glycine derivative of chitosan consists of: activation of the magnetic support with epichlorohydrin, followed by reaction with either glycine to produce the reference material (i.e., Gly sorbent) or glycine ester hydrochloride, followed by hydrazinolysis to synthesize the hydrazide functionalized sorbent (i.e., HGly sorbent). The materials are characterized by titration, elemental analysis, FTIR analysis (Fourrier-transform infrared spectrometry), TGA analysis (thermogravimetric analysis) and with SEM-EDX (scanning electron microscopy coupled to energy dispersive X-ray analysis). The sorption performances for U(VI), Cu(II), and Zn(II) are tested in batch systems. The sorption performances are compared for Gly and HGly taking into account the effect of pH, the uptake kinetics (fitted by the pseudo-second order rate equation), and the sorption isotherms (described by the Langmuir and the Sips equations). The sorption capacities of the modified sorbent reach up to 1.14 mmol U g^−1^, 1.69 mmol Cu g^−1^, and 0.85 mmol Zn g^−1^. In multi-metal solutions of equimolar concentration, the chemical modification changes the preferences for given metal ions. Metal ions are desorbed using 0.2 M HCl solutions and the sorbents are re-used for five cycles of sorption/desorption without significant loss in performances.

## 1. Introduction

The regulations for drinking water but also for wastewater release into the environment are progressively becoming more restrictive, due to the hazardous health effects for human life (accumulation and persistent pollutants) and negative impacts on water bodies and wildlife. Heavy metals are perfect examples of these persistent contaminants that may accumulate in the food chain with potential dramatic effects on animals and humans. Water resources are also becoming critical, making their recycling/reuse an incentive objective in many countries. In addition, the recycling of metal ions in industrial processes (including from urban mines) is also promoted by national or transnational politics for strategic and valuable critical metals [1]. All these reasons may explain the effort to develop alternative and innovative processes for the recovery of metal ions from industrial and mining effluents. Conventional processes such as precipitation [2], solvent extraction [3], and membrane techniques [4] may face limitations to reach the requested levels of decontamination under economic constraints, especially for diluted effluents. Sorption processes are frequently proposed for the treatment of low-concentration solutions using ion-exchange and chelating resins [5,6], impregnated resins [7], and carbon-based sorbents [8]. Biosorption has been proposed as an alternative process, making profit from materials of biological origin [9], such as algal [10,11], bacterial [12] and fungal biomass [13], biopolymers [14,15], cellulose-based materials [16], and agriculture waste products [17,18] for the recovery of metal ions from dilute solutions. The reactive groups present at the surface of these materials may be similar to the functional groups present on synthetic resins. Their sorption performance may be lower than those exhibited by commercial resins but some advantages such as cost, renewable origin, or more environmentally friendly life cycle can compensate [19]. In addition, these materials are relatively versatile: they can be readily modified (a) physically to develop innovative modes of application (reactive filtration/percolation [20], reactive hollow fibers [21]); or (b) chemically by grafting new reactive groups with higher sorption properties, better selectivity, wider pH range of use, and/or better recyclability [22,23].

Chitosan (aminopolysaccharide) is an emblematic example of these biosorbents that has garnered a great deal of attention over the last 40 years. Chitosan is obtained, at the commercial level, by the deacetylation of chitin (one of the major constituents of the exoskeleton of crustaceans). Since the deacetylation is generally incomplete, the polymer is constituted of both d-glucosamine (2-amino-2-deoxy-d-glucopyranose) and n-acetyl-d-glucosamine (2-acetamido-2-deoxy-d-gluco pyranose) associated by β-(1→4) bonds. The presence of numerous hydroxyl groups brings interesting hydrophilic properties, while the amine groups are very efficient for metal binding. Indeed, the free electron doublet on nitrogen from the amine groups contributes to the chelation of metal cations in near-neutral solutions, while its protonation in acidic solutions opens the way for electrostatic attraction/ion exchange interactions with anionic species [15]. The sorption properties are then strictly controlled by the pH, in terms of stability (dissolving in acidic solutions, with the exception of sulfuric acid solutions) and reactivity. Hydroxyl and amine groups have been abundantly used for chemical modification of the materials to change and improve sorption properties. In addition, the poor properties of these materials in terms of porosity and specific surface area requires using small particles or alternative conditioning (such as hydrogels [21]) for reducing the negative impact of diffusion properties on kinetic performance. Reducing the size of sorbent particles enhances sorption kinetics but at the expense of (a) difficulty in the solid/liquid separation at the end of the process in batch systems; or (b) head loss pressure and blocking phenomena, when used in fixed-bed columns. The incorporation of a magnetic core in the biopolymer readily allows solid/liquid separation and this process has been frequently used for designing chitosan micro- or nano-particles with good mass transfer properties [24,25,26,27,28]. The combination of chemical modification of chitosan and magnetite incorporation allows improving sorption performance in terms of sorption levels, kinetics, and processing (solid/liquid separation) [29,30].

The functionalization of chitosan frequently consists of the grafting of amino-acid moieties [22,31]. Indeed, this contributes to increased density of reactive groups, to insert additional functions with higher selectivity or higher affinity (such as sulfur/thiol groups [32]), and to a larger pH-range of applications. Glycine, one of the most simple and cheap amino-acids, is abundantly documented for the chemical modification of chitosan through the activation of the polymer with thionyl chloride, phosphoryl chloride, glutaraldehyde, or epichlorohydrin [31,33]. The present work is based on the chemical modification of magnetic glycine-grafted chitosan particles (considered as the reference material, the so-called Gly sorbent). The modified sorbent (called HGly) is prepared by the grafting of glycine ester material followed by hydrazinolysis to synthesize hydrazide Gly material.

These two materials are first characterized chemically by elemental analysis, titration, and FTIR analysis in order to clarify the chemical structure of the sorbents and the efficiency in chemical modification of the material. SEM-EDX analysis (scanning electron microscopy coupled to energy dispersive X-ray analysis) confirms the binding of heavy metals, including U(VI), Cu(II), and Zn(II). The sorption properties of Gly and HGly are compared through a study of the pH effect, uptake kinetics, sorption isotherms (in single-metal and multi-metal solutions), desorption performance, and sorbent recycling.

## 2. Results and Discussion

### 2.1. Characterization of Sorbents

The weight loss of the sorbents at 650 °C indicates that the weight fraction of the magnetite core changes with the chemical modification of the material: for activated magnetic chitosan (with epichlorohydrin spacer arms) the weight loss is 38.0 ± 0.8%, for the Gly sorbent it reaches 44.8 ± 1.7%, and is up to 53.1 ± 0.7% for the HGly sorbent. As expected, the fraction of magnetite in the sorbent decreases with the chemical modification of the support. The theoretical weight fraction of magnetite (based on the precursors of the magnetic composites) is 50%; this means that there is a loss of organic fraction during the synthesis. Table 1 shows the elemental analysis of the sorbents Gly and HGly (together with intermediary products). The mass of the equivalent monomer unit for the chitosan used in this study (i.e., deacetylation degree of 90.5%) is about 165 g·mol^−1^; this would correspond to a nitrogen mass fraction of 8.48% (6.06 mmol N g^−1^). This could be attributed to the intrinsic amino groups in chitosan. The grafting of glycine allows for substantially increasing the nitrogen content in the Gly sorbent: the N content increased by a factor of 2.5 compared to activated magnetic chitosan. This means that the glycine grafting is almost quantitative on the activated material: based on the supposed chemical route, the substitution of chloride (from the epichlorohydrin spacer arm) with glycine leads to doubling the amount of nitrogen if the reaction is quantitative. The nitrogen content in Gly represents a molar content of 3.19 mmol N g^−1^ (based on elemental analysis). Surprisingly the fraction of nitrogen is slightly greater on the material grafted with esterified-glycine; no explanation was found to justify this slight increase. Hydrazinolysis of the material (to synthesize HGly) allows a further doubling of the nitrogen content onto the esterified-glycine modified support (10.05% against 4.81%, in terms of the element mass fraction; 7.18 mmol N g^−1^ against 3.44 mmol N g^−1^); this is also consistent with the quantitative substitution of the hydrazide on the glycine ester derivative of activated magnetic chitosan particles. The amine content in HGly reaches 7.18 mmol –NH g^−1^ (based on elemental analysis). These values are under evaluated considering the titration analysis: the amine content is evaluated by titration close to 3.89 and 8.12 mmol –NH g^−1^ for Gly and HGly, respectively (variations in the range 0.7–1 mmol N g^−1^ compared to elemental analysis). In any case, the variations in nitrogen content and amine content prove the successfulness of both the grafting of glycine and further hydrazide modification of the sorbent.

The pH-drift method was used for determining the pHZPC of the sorbents (Figure S5, see Supplementary Material). The values are very close for the two sorbents: 7.4 and 7.47 for Gly and HGly, respectively. This means that in acidic solutions the sorbents are systematically protonated. This also means that Gly holds carboxylic groups is not significantly more acidic than HGly that only holds amine groups. Glycine amino-acid bears two reactive groups that have pKa values close to 2.22 and 9.86 for carboxylic acid and amine moieties, respectively [34]. Obviously, the grafting of the amino-acid on chitosan backbone alters its acid-base properties; however, this means that the amine groups are preponderant in the control of acid-base properties; probably due to the presence of the amine groups on the biopolymer backbone. It is noteworthy that the pKa of amine groups in chitosan varies between 6.4 and 6.8 for partially deacetylated chitosan [35]. In the case of hydrazide derivatives the environment of hydrazide core (presence of neighbor electron withdrawing groups) strongly impacts the value of pKa in the range 7.5–10.90 [36]. Hydrazide being at the end of the substitution chain on HGly, the acid-base character is influenced by the first neighbor groups [37].

Figure S6 and Table S1 compare the FTIR spectra and band assignments of glycine and esterified glycine. The results show a new strong band at 1741 cm^−1^ assigned to the stretching C=O of the ester formed, and besides this, other bands of stretching NH, CH, C–N, bending C–H and COO^−^, and wagging and rocking COO^−^ bonds are also observed (shown in the table). Figure S7 and Table S2 show the spectra and band assignments of the different materials synthesized during the preparation of Gly and HGly sorbents (see Supplementary Material). FTIR analysis confirms the chemical modification of the materials. On the spectrum of the magnetic grafted chitosan with the spacer arm (epichlorohydrin), there is a new band at 788 cm^−1^ assigned to the stretching of CH_2_–Cl that emphasizes the success of the grafting process. On Gly and HGly spectra, two strong bands appear (compared to grafted chitosan) at 1626 cm^−1^ and 1628 cm^−1^ that can be assigned to the C=O bond of the carboxylic group and amide, respectively; this confirms the formation of the new functionalized groups on the spacer-arm activated chitosan and on the chemical derivatives.

Figure S8 and Table S3 show the FTIR spectra and the assignments of the main bands for Gly before and after metal sorption (and after metal desorption); similar data are presented for HGly in Figure S9 and in Table S4 (see Supplementary Material).

The decrease and shifts in the intensity of the bands of the carboxylic and amino groups are observed after metal binding: these reactive groups are involved in the interaction of the metal ions with the sorbents. These bands appear once again after elution; this means that the sorption/desorption has a weak effect on the functional groups (limited chemical changes or degradation). Moreover, there are new peaks appearing after metal loading, especially in the range from 430–415 cm^−1^; these bands have already been reported after metal sorption on polymers [38].

SEM observations (not shown) allowed for measuring the size of sorbent particles that ranged between 100 and 400 µm. Figure S10 shows the SEM-EDX analysis of the activated magnetic chitosan particles (with epichlorohydrin arms, a), Gly sorbent (b and d), and HGly sorbent (c and e) before and after metal sorption at pH 5 (with equimolar concentrations of U(VI), Cu(II), and Zn(II)). The EDX analysis confirms the increase of the carbon content with glycine grafting; on the other hand, for the HGly sorbent the chemical substitution by hydrazide on the carboxylic group decreases the fraction of the O element. The binding of metal ions is confirmed by the appearance of U, Cu, and Zn elements: the relative fractions of these elements are increased for HGly compared to Gly; this means that, as expected, the hydrazide grafting substantially improves sorption performance. The Fe element is representative of the magnetite core; EDX analysis is limited to a very thin external layer of the particles, and the relative intensities should be considered as indicative, but the order of magnitude is consistent with the weight loss results.

Thermogravimetric analyses show that the two sorbents first lose about 8% of their mass at 120 °C: this is due to water loss from the materials (identical fractions for Gly and HGly materials representing absorbed and bound water molecules). The profile of weight loss is slightly shifted toward higher temperatures (by 10 to 30 °C, depending on the temperature range). The final weight loss is higher for Gly (56.4%) than for HGly: (51.3%): the residues represent 43.6% and 48.7%, respectively; this is roughly consistent with the magnetic core fraction deduced from the weight loss at 600 °C as determined above (i.e., 44.8% and 53.1%, respectively). The DTG (derivative thermos-gravimetric analysis) figure identifies two degradation steps for HGly (at temperatures of 259.0 °C and 419.8 °C) and three degradation steps for Gly (at 243.1 °C, 321.6 °C, and 427.7 °C). The first step corresponds to the degradation of chitosan (including dehydration of the saccharide ring and depolymerization), and the other steps correspond to the degradation of chemical substituents (including the decarboxylation of grafted carboxylic groups and the decomposition of amine derivatives) [39,40].

### 2.2. pH Effect on Sorption Properties and the Approach of the Sorption Mechanism

**Figure 1:**
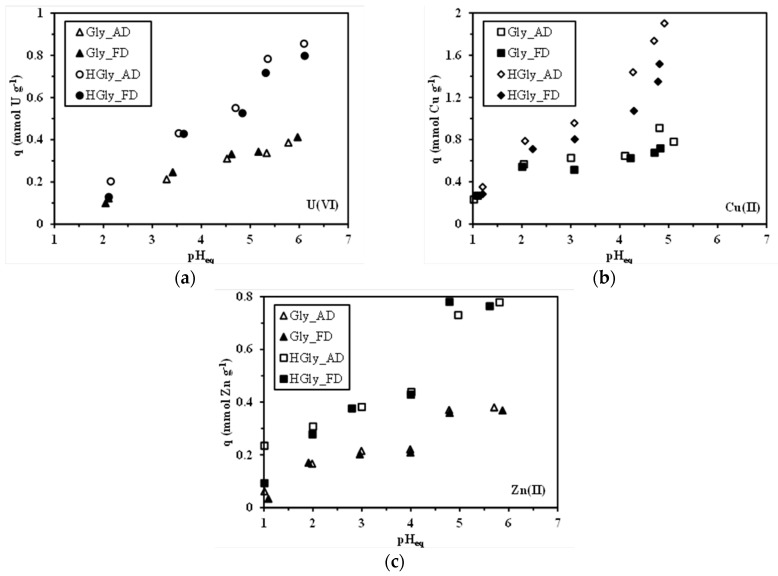
Effect of pH on the sorption of U(VI) (**a**); Cu(II) (**b**); and Zn(II) (**c**) using Gly and HGly sorbents (both the air-dried and the freeze-dried sorbents have been tested for verification of the impact of the drying mode and for the reproducibility of sorption data) (sorbent dosage, SD: 200 mg·L^−1^; *C*_0_: 50 mg U L^−1^; 100 mg Cu L^−1^ and 100 mg Zn L^−1^; T: 20 °C; v: 150 rpm; contact time: 48 h).

The sorption of metal ions is strongly influenced by the pH (Figure 1). The pH may affect metal speciation (through the formation of complexes in the presence of ligands or due to the hydrolysis effect) and then metal affinity of the reactive groups on the sorbent [41]. The pH may also influence the reactivity of functional groups (through their protonation/deprotonation properties). The experiments have been performed on both freeze-dried (FD) and air-dried (AD) materials: the differences are negligible and confirm the reproducibility of the experimental data. The sorption capacities for the three metal ions of the HGly sorbent are higher than those of Gly; this can be directly correlated to the larger density of reactive groups on the hydrazide derivative. The sorption capacities are increasing with the pH. For Gly, the sorption capacities for U(VI) and Zn(II) roughly increase linearly with equilibrium pH, while for Cu(II) the sorption increases from pH 1 to pH 2 and then tends to stabilize; in any case, the sorption capacities are only doubled between pH 1 and pH 5–6 and remain below 0.4 mmol U g^−1^, 0.8 mmol Cu g^−1^, and 0.4 mmol Zn g^−1^. In the case of HGly, the pH has a more marked effect; the grafting of hydrazide allows for reaching sorption capacities as high as: 0.8 mmol U g^−1^, 1.6 mmol Cu g^−1^, and 0.8 mmol Zn g^−1^. In addition, the sorption capacities progressively (but slightly) increase between pH 1 and 4–4.5 before strongly increasing above pH 5.

The determination of pH_ZPC_ for the two sorbents showed that the values are relatively close for Gly and HGly (i.e., 7.47 and 7.40, respectively). In the acidic region the two sorbents are positively charged; the cationic behavior progressively decreases with increasing the pH. The Gly sorbent bears both secondary amines and carboxylic groups while HGly bears secondary amine groups and hydrazide functional groups. Carboxylic groups of (unmodified) glycine are close to 2.32 [42]; this means that they are theoretically deprotonated at pH higher than 2.5. The acid-base properties are modified by grafting on the chitosan-based support but carboxylate groups may contribute to binding metal cations when the pH increases. In the case of chitosan, the pK_a_ of amine groups depends on the deacetylation degree (being in the range from 6.4–6.8 for the most common commercial samples, [35]). Their chemical modification also contributes to changing the value of pKa for these amine groups; however, in acidic solutions (pH of experimental studies: 1–6), the amine groups are mainly protonated. Even with Gly, the amount of amine groups is larger than the amount of carboxylic groups and the global charge of the sorbent is cationic even at the highest pHs, consistently with pH_ZPC_ values. Under these conditions, though a contribution of chelation (on deprotonated amine groups and carboxylate groups) at the mild pH values cannot be rejected, most of the sorption is expected to proceed through ion-exchange/electrostatic attraction on protonated amine groups and carboxylic groups. In the case of HGly, the number of amine functions is obviously increased, enhancing the sorption of metal cations, especially at the highest pH values. Lindgren and Nieman [43] reported the apparent pK_a_s for glycylhydrazide (the end-block of the HGly sorbent) close to 2.38 and 7.69 (for the doubly protonated glycylhydrazide). This may explain the reinforced increase of the sorption above pH 4–4.5: some amine groups on the ending-block are deprotonated and available for the chelation of metal cations. Obviously, the competition effect of protons (in terms of chelation and ion-exchange/electrostatic attraction properties on carboxylic and amine moieties) is stronger at low pH values and the sorption capacities are relatively low while the progressive deprotonation of these reactive groups improves metal binding. Feitoza et al. [44] discussed the sorption of copper on glycine-coated maghemite and correlated the sorption to the balance in charges between sorbent and metal ions: at low pH, the presence of free copper (i.e., Cu^2+^) and positively-charged reactive groups (carboxylic acid and amine groups) causes an electrostatic repulsion and limited metal binding. On the opposite hand while increasing the pH, this repulsion effect is progressively minimized with metal binding occurring between positively-charged nanoparticles and negatively-charged copper hydrolyzed species. The highest sorption occurred at high pH (above 6.5) through both electrostatic attraction and/or chelation on deprotonated amine groups. Table S5 (see Supplementary Materials) shows the distribution of the main metal species under selected experimental conditions (total metal concentration used for pH study). For Cu(II), free Cu^2+^ represents most of the metal in solution (i.e., above 98%) and all the species are cationic; however, at pH 1 about 8% of copper is present under the form of CuCl^+^. The relative stability of the metal as free Cu(II) cations means that speciation is not the controlling step for Cu(II) sorption efficiency; the pH effect is thus mainly affected by the protonation/deprotonation of reactive groups. In the case of Zn(II), similar trends are observed: at pH 1 and 2 small amounts of ZnCl^+^ are observed; but free Zn^2+^ represents the largely predominant form of zinc in the solution. Again the weak variation in metal speciation has a limited impact on the interpretation of the pH effect on Zn(II) removal efficiency: the predominating effect is associated to the charge effect on the reactive groups. These cationic species will be preferentially bound under conditions enhancing the deprotonation of reactive groups. A completely different trend is observed in the case of uranyl. Table S5 shows that in strong acidic solutions (i.e., pH 1 and 2) sulfate complexes (neutral (UO_2_SO_4_), and to a lesser extent anionic form, (UO)_2_(SO_4_)_2_^2−^) predominate. Anionic species could be bound on protonated groups; however, the fraction of this anionic species is lower than 14% at pH 1 (and even lower at pH 2); the strong competition of counter-anions (brought by the acidification of the solution) limits the sorption efficiency. When the pH increases, the fraction of free and polynuclear uranyl cations (UO_2_^2+^, (UO_2_)_3_(OH)_5_^+^, and (UO_2_)_4_(OH)_7_^+^, the most representative polynuclear and polyhydrolyzed species) strongly increases, and these species will be efficiently bound to deprotonated amine groups and carboxylate groups. In the case of uranyl, both the speciation of the metal and the charge of the reactive groups may contribute to explaining the increase in sorption efficiency. The binding of uranyl under the form of polynuclear species leads to a significant increase in metal recovery since the binding of one polynuclear uranyl corresponds to the binding of 2 or 3 moles of uranium. In an attempt to correlate uranyl sorption to metal speciation, Guibal et al. [45] plotted the sorption isotherms of U(VI) on chitosan at different pHs as a function of the total concentration of polynuclear hydrolyzed species (instead of total uranyl concentration). They successfully showed, in this case, that the sorption isotherms are very favorable (almost irreversible) while the conventional plot (as a function of total metal concentration) was unfavorable and only becomes favorable when the metal concentration reaches a given concentration (pH-dependent) corresponding to the appearance of polynuclear hydrolyzed species. Similar trends were found in the case of V(V) sorption on chitosan [46]. Table S6 (see Supplementary Materials) shows the metal distribution at pH 5 for increasing metal concentrations (in the range used for sorption isotherms). Similar conclusions are raised for Cu(II) and Zn(II): free metal species predominate over the 1–5 mM concentration range. In the case of U(VI), the polynuclear hydrolyzed species predominate on the complete range of concentration and their relative fraction increases with the total metal concentration: the sorption progressively becomes more favorable due to the increasing predominance of polynuclear hydrolyzed species.

In addition to the effect of pH on the sorption capacities, it is also important to consider pH changes during the sorption. Figure S11 (see Supplementary Material) shows different trends for U(VI), Cu(II), and Zn(II). In the case of Zn(II) sorption, the equilibrium pH is close to the first bisector and the only remarkable change concerns a slight decrease in the pH for initial pH values higher than 5. In the case of Cu(II), the equilibrium pH remains close to the first bisector until pH 4, and at higher pH the equilibrium value significantly decreases (by more than 1 pH unit at pH 6); this is probably due to the formation and binding of hydrolyzed species. A completely different behavior is observed for U(VI) sorption. Even at initial pH 3 (and up to pH_0_: 5) a substantial increase of the pH is observed by 0.5–0.8 pH units, while the pH stabilizes to the initial value only at pH 6. While Cu(II) and Zn(II) are forming “simple” hydroxide species (such as Cu(OH)^+^, Cu(OH)_2_, or Zn(OH)^+^ and Zn(OH)_2_ under the concentration and pH conditions selected in this work) when the pH increases, in the case of U(VI) polynuclear hydrolyzed species may be formed (such as (UO_2_)_2_(OH)_2_^2+^, (UO_2_)_3_(OH)_5_^+^, apart from the mononuclear species: (UO_2_)OH^+^, [47]): the predominance of the metal species depends on both the pH and the metal concentration [48]. The pH increase may be attributed to the binding of protons and/or the release of hydroxide ions (by displacement of uranyl speciation and/or ion-exchange of metal hydrolyzed species during sorption).

In the case of uranyl-glycine chelation, Su et al. [49] reported that the conformation of the complex changes with temperature, pH, and other environmental parameters. At room temperature, five-coordination in the equatorial plane of uranyl is enhanced. In neutral and acidic solutions, they suggest that one or two glycine molecules bind as zwitterions offering their bidentate carboxylic groups (while water molecules occupy the remaining space in the first coordination sphere). Obviously the presence of other potential reactive groups on the modified arm (arm spacer groups, including secondary amine groups and OH groups) may also contribute to binding metal ions on the same chain or on the vicinal chain to comply with the expected coordination sphere. Bi et al. [50] proposed different mechanisms for the complexation of Cu(II) in solution with glycine and on its binding on both glycine-grafted silica gel and ion-imprinted silica gels functionalized with glycine, diglycine, and triglycine. The spatial arrangement (free or controlled by the ion imprinted) and the intermolecular distances influence the mode of interaction between copper ions and carboxylic groups and/or amine groups (mono-functional groups or di-functional group interactions). While only carboxylate groups are involved, the charge neutralization controls copper binding on a 2:1 (COO^−^/Cu(II)) mode with vicinal glycine groups; for systems involving both carboxylate and amine groups, Cu(II) binds with two secondary amine groups and two carboxylic groups. In the case of diglycine decorated silica gel, the mechanism may in addition consist of the direct binding of Cu(II) with four nitrogen sites (coming from two vicinal diglycine chains). The presence of hydrazide groups in HGly offers additional groups that may involve amine groups from vicinal chains: both amine groups of chitosan, of the glycine intermediary substituent, and of the hydrazide moiety can take part (at different extents to metal binding).

### 2.3. Uptake Kinetics

**Figure 2:**
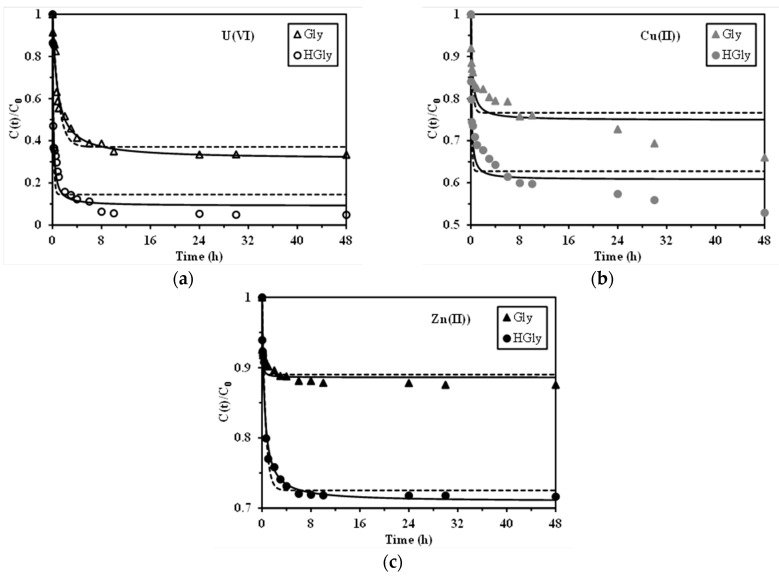
Uptake kinetics for U(VI) (**a**); Cu(II) (**b**); and Zn(II) (**c**) sorption using Gly and HGly sorbents at pH_0_: 5 (*C*_0_: 20 mg U L^−1^; 25 mg Cu L^−1^ and 30 mg Zn L^−1^; SD: 305 mg·L^−1^ for Gly/U, 183 mg·L^−1^ for HGly/U, 310 mg·L^−1^ for Gly/Cu; 200 mg·L^−1^ for HGly/Cu; 300 mg·L^−1^ for Gly/Zn and 350 mg·L^−1^ for HGly/Zn; T: 20 °C; v: 150 rpm) (dashed lines: modeling with the PFORE (pseudo-first order rate equation); solid lines: modeling with the PSORE) (pseudo-second order rate equation).

Figure 2 shows the uptake kinetics for the sorption of U(VI), Cu(II), and Zn(II) using both Gly and HGly. For U(VI) and Zn(II), the sorption kinetics follow the same trend: (a) strong initial sorption for the first two hours of contact that represents between 73% and 89% of the total sorption at equilibrium; and (b) a slower sorption phase that lasts for 10–24 h. The initial sorption step counts for the sorption on external reactive groups (at the surface and within the first layers of the polymer coating), while the second step may consist of the diffusion of metal species within the modified polymer layer (coating the magnetic core) and possibly is accompanied by the proper swelling kinetic of this layer. This second phase is representing a limited (and in some cases almost negligible) fraction of the total sorption. These sorbents have a particle size ranging between 100 and 400 µm; this means that taking into account the magnetic core, the organic compartment represents a thin layer and the fraction of the sorption impacted by the intraparticle diffusion is rather limited. This confirms the interest of synthesizing small particles with a magnetic core for limited resistance to intraparticle diffusion and ready solid/liquid separation. In the case of Cu(II) sorption, the trend is slightly different; three phases can be identified: (a) a first initial strong and fast sorption (within the first 30 min of contact, 52–56% of total sorption at 48 h of contact); (b) an intermediary with a much slower kinetic rate that lasts for 8 h and represents an additional sorption of 30% of the total binding; and finally (c) a slow phase that probably did not reach equilibrium after 48 h of contact. The different behavior observed for Cu(II) sorption may be associated to the slight formation of hydrolyzed species of copper, which, in turn, may affect the diffusion and binding of these copper species.

The kinetic profiles have been modeled using the pseudo-first order rate equation (PFORE, dashed lines in Figure 2) and the pseudo-second order rate equation (PSORE, solid lines in Figure 2). Table 2 reports the parameters of the two models when applied to kinetic profiles. The estimated variances allow for confirmation in most cases of the better fit of experimental data with the PSORE, at least for U(VI) and Zn(II) sorption. Another parameter that contribute to evaluating the quality of the fit is the comparison of the calculated values of the sorption capacities at equilibrium (*q_eq,calc_*) with the corresponding experimental value (i.e., *q_eq,exp_*). In most cases the PSORE allows for approaching the experimental value with more accuracy (less than 8%) than the PFORE model (up to 12%). In the case of Cu (II) sorption, both PFORE and PSORE show a relatively bad fit of the experimental data. This is directly correlated to the fact that the equilibrium is not formally reached due to the mechanisms of hydrolysis that contribute to limiting the transfer rate (effects of the hydrolysis kinetic rate, resistance to intraparticle diffusion, and/or formation of hydrolyzed colloids). In the case of PFORE the apparent kinetic rate, k_1_, increases for the Gly sorbent following the sequence: U(VI) < Cu(II) < Zn(II) (value for Cu(II) should be considered as an approximation of the order of magnitude) from 0.017 to 0.32 min^−1^. In the case of the HGly sorbent, the variation is rather limited (between 0.108 and 0.103 min^−1^). For the PSORE, the trends are completely different: the apparent kinetic rate, k_2_, increases from 0.13 to 26 g·mmol^−1^·min^−1^ for Gly for the series: U(VI) < Cu(II) < Zn(II), while for HGly the ranking followed the reciprocal trend with a rather limited variation between 0.012 and 0.04 g·mmol^−1^·min^−1^. Though the experimental conditions are not strictly identical, it is noteworthy that the HGly sorbent is less influenced in terms of kinetic rate by the nature of the metal, at least compared to the Gly sorbent. It is difficult to rank the two sorbents in terms of kinetic criterion due to changes in their response to the type of metal ions. The simplified modeling of kinetic profiles does not identify the contribution of diffusion mechanisms, which may be influenced by metal speciation (formation of hydrolyzed species and polynuclear species) that, in turn, strongly impacts the size of metal ions, their charge, and their mass transfer.

### 2.4. Sorption Isotherms

**Figure 3:**
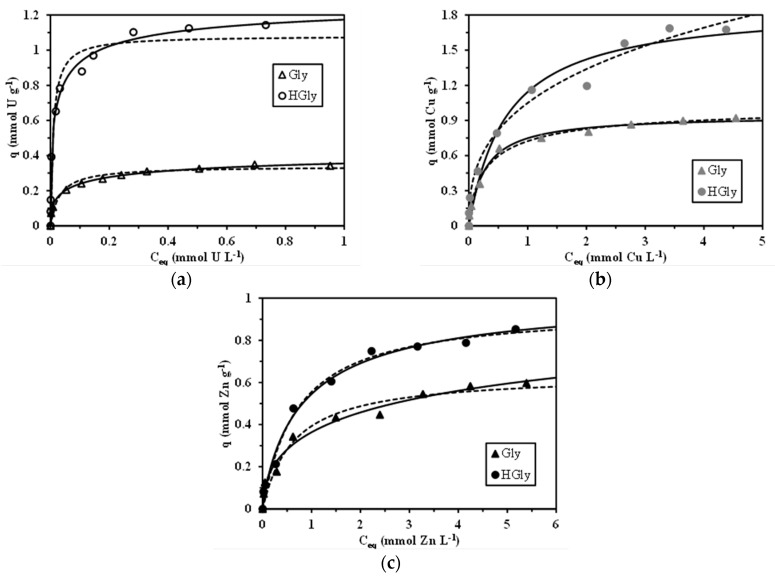
Sorption isotherms for the recovery of U(VI) (**a**); Cu(II) (**b**); and Zn(II) (**c**) using Gly and HGly sorbents at pH_0_: 5 (dashed lines: modeling with the Langmuir equation; solid lines: modeling with the Sips equation) (pH: 5; T: 20 °C, v: 150 rpm; time: 48 h; SD: 300 mg·L^−1^ for U(VI), 350 mg·L^−1^ for Cu(II), and 500 mg·L^−1^ for Zn(II)).

The sorption isotherms are reported in Figure 3 for the three metals ions and the two sorbents. The sorption isotherms are characterized by two specific criteria: (a) the maximum sorption capacity obtained on the saturation plateau and (b) the affinity coefficient (which is proportional to the initial slope). These two parameters are also correlated to the parameters of the Langmuir equation (the sorption capacity at saturation of the monolayer, and the affinity coefficient). The sorption isotherms in Figure 3 are modeled using the Langmuir (dashed lines) and the Sips equations (solid lines) with the parameters reported in Table 3.

The comparison of estimated variances shows that the Sips model fits the experimental data better than the Langmuir equation; obviously increasing the number of parameters in the equation allows for reaching a better simulation of the isotherm profiles. The statistical analysis (estimated variances) is improved but at the expense of a loss of physical significance. For example, the comparison of the maximum sorption capacities of the two models with experimental values shows that the Sips model strongly overestimates the maximum sorption capacities while the values for the Langmuir equation are much closer to the experimental values. For U(VI), the maximum sorption capacity increases from 0.349 to 1.14 mmol U g^−1^ after Gly functionalization. The chemical modification of the Gly sorbent by hydrazide grafting significantly improves the sorption capacity for the three metal ions. However, the impact has an increasing impact on the following sequence: U(VI) > Cu(II) > Zn(II). The maximum sorption capacity (experimental values) reached for the Gly sorbent is 0.349 mmol U g^−1^, 0.920 mmol Cu g^−1^, and 0.598 mmol Zn g^−1^. Compared to the amine content of this material (i.e., 3.44 mmol N g^−1^ by elemental analysis and 3.89 mmol –NH g^−1^ by titration) it is not possible to identify a stoichiometric ratio that could fit with the expected sorption mechanisms (either ion-exchange/electrostatic attraction or chelation) even taking into account the possible contribution of carboxylic groups (which should represent about 1.7–1.9 mmol COO^−^ g^−1^, based on the suggested structure of the sorbent and the quantitative grafting of glycine onto the chitosan backbone, see above). In the case of the HGly sorbent the maximum sorption capacities are increased to 1.14 mmol U g^−1^ (3.27 times the reference value of Gly), 1.69 mmol Cu g^−1^ (1.84 times), and 0.853 mmol Zn g^-1^ (1.43 times). The amine content of HGly varies between 7.18 mmol N g^−1^ (elemental analysis) and 8.12 mmol –NH g^−1^ (titration); this means an increase (compared to Gly) of 3.74–4.23 mmol·g^−1^ (depending on the analytical method). Again, the stoichiometric ratio is difficult to establish: the co-existence of different metal species also contributes to explaining this difficulty. The highest increase observed for U(VI) sorption can probably be associated to the binding of polynuclear uranyl species, which are predominating at pH 5 or higher. The comparison of affinity coefficients (with both the Langmuir and Sips equations) shows that the sorbents have higher affinity for U(VI) (especially HGly) compared to Cu(II) and Zn(II). This can be explained by the Pearson’s rules (hard and soft acid base theory, HSAB) [51,52]: hard acids react fast and in priority with hard bases. Uranyl is classified as a hard acid (softness parameter: −0.27) while Cu(II) (+0.38) and Zn(II) (+0.35) are classified among the borderline metal ions. Amine and hydrazide-based groups are considered as hard bases (though softer than the negatively charged O donor atoms, such as those found in carboxylate reactive groups [53]): these functional groups may have a preference for U(VI) over Cu(II) and Zn(II).

The sorption capacities of Gly and HGly for U(VI), Cu(II), and Zn(II) are compared to those obtained in the literature with other sorbents and biosorbents under similar pH conditions (Table 4). In most cases the sorption properties of the Gly sorbent are comparable to those obtained with other biopolymers, and the grafting of hydrazide brings sorption properties that are among the highest of the selected materials. Though some specific materials may exhibit higher performance: for example, semi-IPN hydrogel (semi-interpenetrating polymer network) based on chitosan and gelatin for Cu(II) recovery [54] or magnetic peptide resin for Zn(II) removal [55]. Though carboxylic groups (in minority compared to amine groups) in the Gly sorbent can bring different affinities for metal cations (according to the HSAB rules), the large increase in sorption capacities observed against chitosan (and compared to other conventional sorbents) can probably be attributed to the much higher density of reactive groups (amine groups) that have a good affinity for metal cations in mild acidic or neutral conditions (deprotonation of amine groups) through chelation on lone electron pairs. This is especially important in the case of the HGly sorbent (see Table 1, nitrogen content).

### 2.5. Sorption in Multi-Metal Solutions—Selectivity Study

**Figure 4:**
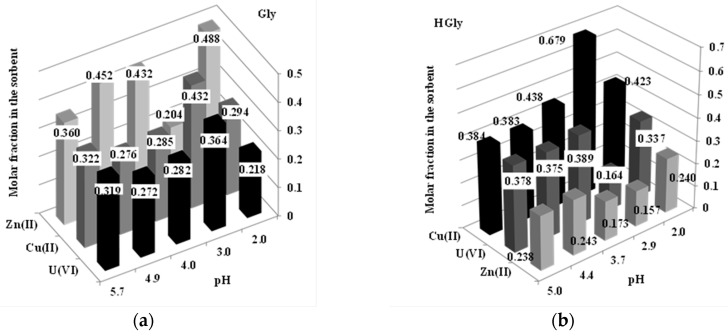
Molar fractions of Cu(II), U(VI), and Zn(II) in the sorbent (Gly (**a**) and HGly (**b**)) for multi-metal solutions (equimolar concentrations of the three metal ions) at different equilibrium pH values (number experimental values) (*C*_0_: 0.3 mmol·L^−1^; T: 20 °C; v: 150 rpm; time: 48 h; SD: 2 g·L^−1^ for Gly and 1 g·L^−1^ for HGly).

Though the comparison of affinity coefficients concluded that the sorbents (and more specifically HGly) have a stronger interaction with U(VI) a complementary experiment was carried out with multi-metal solutions (containing equimolar concentrations of U(VI), Cu(II), and Zn(II); i.e., 0.3 mM) at different pH values in order to verify the sorption selectivity. Figure 4 plots, for the two sorbents, the molar fractions of the different metal ions at different values of equilibrium pH (in the range from 2–5.7). The enrichment or depletion of metal ions in the sorbents are not very marked (compared to the initial metal ratio that was close to 0.33 in the solution). The most remarkable results are the slight enrichment of Zn(II) on the Gly sorbent more specifically at pH 2, 4, and 4.9, while U(VI) and Cu(II) appear to be slightly less sorbed (molar fractions being in most cases below 0.3), except at pH 3. In the case of the HGly sorbent, the trends are reversed: Zn(II) is strongly depleted while Cu(II) removal is significantly enhanced and U(VI) recovery is maintained at mild levels (i.e., consistent with the initial molar fraction of the metal in the solution). At pH 2.9, HGly has a marked preference for Cu(II) over the two other metal cations: this is the unique system (pH 2.9 and HGly sorbent) that allows a substantial enrichment of the sorbent in terms of metal separation. However, these results show that the sorbents cannot be applied for selective separation of U(VI), Cu(II), and Zn(II), at least in terms of sorption properties. This selectivity could be improved by the desorption step.

### 2.6. Metal Desorption and Sorbent Recycling

**Figure 5:**
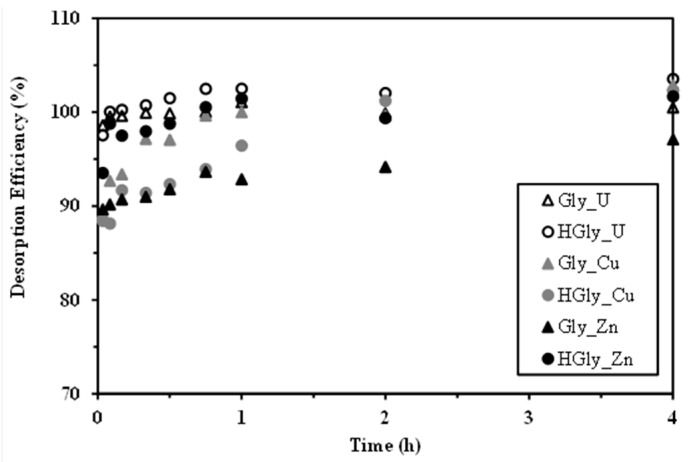
Desorption kinetics for the recovery of U(VI), Cu(II), and Zn(II) from metal loaded Gly and HGly sorbents (loaded sorbents were recovered from uptake kinetics, see experimental conditions in Figure 2; desorption operated using 0.2 L of 0.2 M HCl solutions for Cu(II) and Zn(II) experiments and 0.15 L/0.16 L for Gly and HGly, respectively, for U(VI) experiments).

The metal-loaded sorbents have been used for investigating the desorption efficiency using a 0.2 M HCl solution. The first step consisted of investigating the kinetics of desorption (Figure 5) (with the sorbents collected at the end of the uptake kinetic experiments). Metal desorption is relatively fast (at least compared to the uptake kinetics): 1 h of contact is sufficient to reach equilibrium and/or the complete elution of the metal ions, regardless of the metal and the sorbent. Except for the Zn(II)-Gly system for which the desorption efficiency does not exceed 95%, for the other systems the metal desorption is complete. It is noteworthy that a focus on the first few minutes of contact (not shown) confirms that the first 2 min of contact is sufficient for achieving about 98% of U(VI) desorption, while 92% of Zn(II) and 90% of Cu(II) are eluted within the same contact time.

The sorbents were tested for a series of 5 cycles of sorption and desorption to evaluate the feasibility of sorbent recycling. Table 5 reports the efficiencies of sorption and desorption during the 5 cycles. Under selected intrinsic conditions (which were maintained identical for the 5 cycles) the sorption efficiencies tend to slightly decrease with the number of cycles. However, this decrease does not exceed 6–9% for U(VI), 12–20% for Cu(II), and about 9% for Zn(II) at the last cycle. In the case of U(VI), the desorption efficiency remains stable over the five cycles; this may explain that the loss in sorption efficiency is also minimized compared to other metal cations. In conclusion, these sorbents have relatively good recycling properties and the sorbents can be re-used for a minimum of five cycles with a limited decrease in sorption/desorption performance.

## 3. Materials and Methods

### 3.1. Materials

Chitosan (deacetylation degree: 90.5%), glycine, and thionyl chloride were supplied by Sigma-Aldrich (Taufkirchen, Germany) and were used as received without purification. Ethanol and epichlorohydrin were purchased from Fluka AG (Buchs, Switzerland). Metal salts (i.e., CuCl_2_∙2H_2_O, ZnCl_2_, and uranyl sulfate) were obtained from Sigma-Aldrich. Stock metal solutions were prepared at the concentration of 1 g L^−1^ and were diluted with Milli-Q water at a fixed pH value just before use. Other reagents were supplied by Prolabo (VWR, Fontenay-sous-Bois, France).

### 3.2. Synthesis of Sorbents

#### 3.2.1. Synthesis of Magnetite-Chitosan Micro-Particles

The incorporation of the magnetite core into chitosan particles was obtained by a hydrothermal co-precipitation method (the so-called Massart method, [82]). Chitosan (4 g) was dissolved in 200 mL of (20%, w/w) acetic acid solution before adding 6.62 g of FeSO_4_∙7 H_2_O and 8.68 g of FeCl_3_ (to respect a 1:2 molar ratio between Fe(II) and Fe(III) salts, and a 1:1 theoretical ratio between the magnetite based solid and chitosan). The mixture was precipitated at 40 °C by dropwise addition of NaOH (2 M) under constant stirring to adjust the pH to 10–10.4. The mixture was maintained under agitation for 1 h at 90 °C. The material was then collected by decantation and magnetic separation. Several rinsing steps were operated to remove unreacted reagents. This wet product was then added to an alkaline solution of epichlorohydrin (0.01 M in 0.067 M NaOH, corresponding to pH 10); the proportion was 1:1 between the crosslinking agent and the magnetic chitosan support. The mixture was maintained under agitation for 2 h at 40–50 °C. Unreacted epichlorohydrin was removed by several washing steps. The procedure is described in Figure S1 (see Supplementary Material).

#### 3.2.2. Synthesis of Activated Magnetite-Chitosan Micro-Particles

The activation of magnetic chitosan particles (grafting of spacer arms) consisted of the reaction of the material dropped into 150 mL of ethanol/water solution (1:1, v/v) prior to the addition of 15 mL of epichlorohydrin. The suspension was maintained under reflux for 3 h. After filtration, and washing with ethanol (3 times) and Milli-Q water, the material was collected by decantation and magnetic separation (Figure S1, see Supplementary Material).

#### 3.2.3. Synthesis of Glycine Ester Hydrochloride

Glycine (7.5 g, 0.1 mole) was added to 100 mL of ethanol. The mixture was cooled to −10 °C before adding 7.9 mL (0.11 mole) of thionyl chloride dropwise. The reaction was performed under stirring at −5 °C for 3 h. The mixture was then maintained at room temperature for 24 h before the extraction of the solvent under vacuum. An additional washing step with absolute ethanol was performed and re-evaporated. The synthesis yields 91% (11.3 g) and the melting point of the product was close to 178 °C. The reaction is described in Figure S2 (see Supplementary Material).

#### 3.2.4. Synthesis of Gly Sorbent and Glycine-Ester Magnetic-Chitosan Particles

The activated magnetite-chitosan particles (produced in Section 3.2.2) were introduced into ethanol in the presence of either 16 g of glycine or 22.2 g of glycine ester hydrochloride. The pH of the suspension was adjusted to 9.5–10 with 1 M NaOH solution and the suspension was refluxed for 6 h. The final product was separated by decantation and magnetic separation and then washed three times with ethanol and Milli-Q water. The stock was divided in two parts that were respectively air-dried at 45 °C for 5 h and freeze-dried for 24 h. Figure S3 (see Supplementary Material) shows the schematic route for the synthesis of the Gly sorbent, while Figure S4 shows the synthesis route for the glycine-ester magnetic-chitosan particles (continued with the final synthesis of HGly).

#### 3.2.5. Synthesis of HGly

Glycine-ester magnetic-chitosan particles (air-dried stock) were dropped into 20 mL of absolute ethanol and 30 mL hydrazine hydrate (60%, *w*/*w*). The mixture was maintained under reflux for 4 h. The collected material was washed several times with ethanol and Milli-Q water before being dried at 45 °C for 5 h. The same procedure was used with the freeze-dried stock but the final drying was performed with the freeze-drier (−54 °C, 0.1 mPa) for 24 h. Figure S4 (see Supplementary Material) shows the schematic route for the synthesis of HGly.

### 3.3. Characterization of Sorbents

The elemental composition of the sorbents (C, H, and N percentage composition) was determined using an automatic element analyzer (CHNOS Vario EL III elemental analyzer, Elementar Analysensysteme GmbH, Sonaustraβe, Germany). The FTIR spectra of the different materials (synthesized sorbents at the different stages of the synthesis, before and after metal sorption and after metal desorption) were obtained using a FTIR-ATR spectrometer (Attenuated Total Reflectance tool connected to a Bruker VERTEX 70 spectrometer, Bruker Optik GmbH, Ettlingen, Germany). The magnetite content in the sorbent was determined by weight loss at 650 °C. The content in the amine groups were estimated by volumetric titration. Thirty mL of 0.05 M HCl solution (*C_HCl,_*_1_) were added to 0.1 g of material under mixing for 15 h. The residual concentration of HCl (*C_HCl_*_,2_) was titrated with 0.05 M NaOH solution in the presence of phenolphthalein as the pH indicator. The concentration of amine groups was obtained using the following equation:

(1) $$\left(-{\mathrm{NH}}_{2}\right)=\frac{\left(C_{HCl,1}-C_{HCl,2}\right)\times 30}{0.1}$$

The pH_ZPC_ (pH of zero-charge) was determined by the so-called pH-drift method. A given (identical) amount of sorbent (i.e., 100 mg) was distributed into 50 mL of a 0.1 M NaCl solution at fixed initial pH range (pH_0_, varying between 2.0 and 11.0). The suspension was maintained under agitation in closed flasks for 48 h. The final pH (pH_f_) was monitored and compared to the initial pH value. The pH_ZPC_ corresponds to pH_0_ = pH_f_.

The morphology and size of the sorbent particles and the EDX analysis of the samples were obtained using a scanning electron microscope coupled with an energy dispersive X-ray diffraction analyzer: Quanta FEG 200 (FEI France, Thermo Fisher Scientific, Merignac, France) equipped with an Oxford Inca 350 EDX microanalyzer (Oxford Instruments France, Saclay, France). Thermogravimetric analyses were performed using a Pyris 1 TGA thermogravimetric analyzer (Perkin Elmer, Villebon-sur-Yvette, France); samples were maintained for 3 min at 30 °C under air atmosphere (flow rate: 60 mL·min^−1^, 33% O_2_) before being analyzed between 30 °C and 900 °C (temperature ramp: 10 °C·min^−1^; flow rate: 60 mL·min^−1^, 33% O_2_).

### 3.4. Sorption Studies

Sorption studies were performed in batch systems. The solutions were prepared by dilution of stock metal solutions and the pH of the solutions was controlled with 0.1 M or 1 M HCl or NaOH solutions using a Cyber Scan pH 6000 (Eutech Instruments Pte, Ltd., Nijkerk, The Netherlands). The pH was not controlled during the sorption process but it was systematically monitored at the end of the experiment in order to make a diagnosis on the possible mechanisms of metal precipitation. Some experiments were duplicated or triplicated in order to evaluate the reproducibility: the overall variation did not exceed 6%.

The experiments were performed by mixing a given amount of sorbent (m, g) with a fixed volume of solution (V, L; in most cases 100-mL volumes for “equilibrium” experiments and 1-L volumes for uptake kinetics) at fixed pH values and variable initial metal concentrations (*C*_0_, mg·L^−1^ or mmol·L^−1^). The sorbent dosage varied with the experiments (between 200 and 500 mg·L^−1^). The pH was varied between 1 and 6 for the investigation of the pH effect, and at pH 5 for other experiments. The initial metal concentration was varied in the ranges from 5–250 mg U L^−1^, 5–300 mg Cu L^−1^, and 5–370 mg Zn L^−1^ for the study of sorption isotherms. Sorption experiments (and more generally “equilibrium” experiments) were performed on a reciprocal shaker (agitation speed: 150 rpm) while uptake kinetics were carried out in a jar-test (glass-blade agitator at 140 rpm). After the required contact time (48 h for equilibrium studies, or varying contact time for the study of uptake kinetics) samples were collected, filtrated on a membrane (1 µm pore size) and analyzed for residual metal concentration (*C_eq_*, mg·L^−1^ or mmol·L^−1^) using an inductively coupled plasma atomic emission spectrometer (ICP-AES, Activa M, Horiba France, Longjumeau, France). The sorption capacity (*q*, mg·g^−1^ or mmol·g^−1^) was calculated by the mass balance equation: *q* = (*C*_0_ − *C_eq_*)V/m.

For the desorption study (and sorbent recycling) similar procedures were used. The eluent was HCl (0.2 M). Since the desorption was faster, a shorter contact time was selected (after testing the desorption kinetics). A rinsing step was systematically integrated in the management of the sorption/desorption cycles. The mass balance equation was systematically used for calculating sorption and desorption efficiencies (and/or capacities). The magnetic separation allows for recovering the quasi totality of sorbent particles and the mass loss did not exceed 6% at the end of the fifth cycle.

Note: Since the actual experimental conditions vary with the sorbent and the metal, and in order to avoid the confusing editing of experimental conditions in this section, the detailed experimental conditions are systematically reported in the captions of the figures.

### 3.5. Modeling of Uptake Kinetics and Sorption Isotherms

The experimental results were modeled using conventional equations such as the pseudo-first order rate equation (PFORE, Equation AM1) and the pseudo-second order rate equation (PSORE, Equation AM2), while the sorption isotherms were fitted by the equations of Langmuir (Equation AM3) and Langmuir-Freundlich (the so-called Sips model, Equation AM4). These models are reported in the Supplementary Material.

## 4. Conclusions

The successful grafting of hydrazide onto magnetic glycine-functionalized chitosan allows for enhancing the sorption properties of the sorbent for U(VI), Cu(II), and Zn(II). Optimum sorption occurs at a pH close to 5: the deprotonation of carboxylic acid on the Gly sorbent and the progressive decrease of the competitor effect of protons for binding on amine-based reactive groups on both Gly and HGly may explain this increase in sorption properties. This is also controlled by the speciation of metal ions, including their ability to form polynuclear hydrolyzed species for U(VI). Sorption isotherms are fitted by the Langmuir and Sips equations. The increase in maximum sorption capacity due to chemical functionalization of the Gly sorbent depends on the metal: the enhancement in the sorption property follows the series U(VI) > Cu(II) > Zn(II). Uptake kinetics are relatively fast and the kinetic profiles are well fitted by the pseudo-second order rate equation for U(VI) and Zn(II): U(VI) removal occurs faster than Zn(II). In the case of Cu(II) uptake, the probable occurrence of slow mechanisms of hydrolysis may explain the slower kinetic rates. Metal desorption is successful using the 0.2 M HCl solutions: kinetics of desorption are faster than uptake kinetics and the sorption and desorption properties are slightly reduced during 5 cycles of sorption/desorption.

SEM-EDX, titration, elemental analysis, TGA analysis and FTIR analysis have been used to characterize the structure and composition of the sorbents and for approaching the synthesis routes and the mechanisms of interaction with metal ions: amine-based groups are involved in metal binding though carboxylate groups and may also contribute in the case of the Gly sorbent. The mechanisms of electro-static attraction and chelation can be simultaneously or successively involved depending on the pH and the speciation of metal ions.

## Figures and Tables

**Table 1:** Elemental analysis of the sorbents Gly and HGly (together with activated magnetic chitosan and esterified-glycine intermediate).

Material	C (%)	H (%)	N (%)
Activated magnetic chitosan	15.43 (±0.33)	2.61 (±0.15)	1.79 (±0.03)
Gly sorbent	19.41 (±0.35)	2.82 (±0.01)	4.46 (±0.03)
Esterified Gly sorbent	27.95 (±0.04)	2.95 (±0.04)	4.81 (±0.05)
HGly	23.74 (±0.61)	4.03 (±0.12)	10.05 (±0.07)

**Table 2:** Uptake kinetics for U(VI), Cu(II), and Zn(II) recovery using Gly and HGly sorbents—Modeling with the PFORE and the PSORE.

Sorbent	Metal	*q_eq,exp_*	*q_eq,calc_.*	*k*_1_ × 10^2^	*EV* × 10^4^	*q_eq,calc_.*	*k*_2_ × 10^1^	*EV* × 10^4^
		PFORE				PSORE		
Gly	U(VI)	0.166	0.157	1.73	1.44	0.171	1.28	1.12
	Cu(II)	0.429	0.295	6.26	41.2	0.318	2.36	27.9
	Zn(II)	0.192	0.169	32.4	4.93	0.175	26.2	2.58
HGly	U(VI)	0.404	0.363	10.8	16.2	0.386	4.01	6.41
	Cu(II)	0.842	0.666	13.0	104.2	0.701	2.41	58.3
	Zn(II)	0.375	0.363	11.7	7.38	0.384	1.17	5.84

*q_eq_*: mmol g^−1^; *k*_1_: min^−1^; *k*_2_: g mmol^−1^ min^−1^; *EV*: estimated variance.

**Table 3:** Sorption isotherms for U(VI), Cu(II), and Zn(II) recovery using Gly and HGly sorbents—Modeling with the Langmuir and the Sips equations.

Sorbent	Metal	*q_m,exp_*	*q_m,L_*	*b_L_*	*EV* × 10^3^	*q_m,S_*	*b_S_*	*n*	*EV* × 10^3^
		Langmuir Equation				Sips Equation			
Gly	U(VI)	0.349	0.338	32.8	0.674	0.474	2.98	2.14	0.064
	Cu(II)	0.920	0.946	3.78	1.305	1.03	2.33	1.29	1.16
	Zn(II)	0.598	0.641	1.59	1.95	1.23	0.418	2.01	1.09
HGly	U(VI)	1.14	1.082	106.9	70.9	1.32	7.86	1.93	1.41
	Cu(II)	1.69	1.878	1.56	16.1	10.2	0.114	2.52	9.29
	Zn(II)	0.853	0.952	1.40	1.35	1.03	1.13	1.16	1.67

*q_m_*: mmol·g^−1^; *b*: L·mmol^−1^; *n*: dimensionless; *EV*, estimated variance.

**Table 4:** Sorption properties for U(VI), Cu(II), and Zn(II) by different sorbents.

Sorbent	pH	*q_m_* (mg·g^−1^)			Ref.
		U(VI)	Cu(II)	Zn(II)	
Cysteine-chitosan magnetic nano-based particles	4	101			[56]
Alanine-chitosan magnetic nano-based particles	4	88			[27]
Functionalized mesoporous carbon	4	97			[57]
Phosphorus-modified resin	5	89			[58]
Cross-linked chitosan	3	74.0			[59]
Merrifield chloromethylated resin anchored with semicarbazone moiety	6.5	48.7			[60]
Ion-imprinted magnetic chitosan resins (IMCR) -glutaraldehyde	5	187.3			[61]
Chitosan-succinate (CS) imprinted polymers	7		47.6		[62]
Amino-modified Fe_3_O_4_	5.2		12.4		[63]
m-PAA-Na-coated MNPs (magnetic nanoparticles)	8		30.0		[64]
Sawdust	5.2		8.1		[65]
Rice husk	5.2		31.9		[66]
Chitosan–zeolite composites	5		14.8		[67]
Semi-IPN hydrogel based on chitosan and gelatin	5.5		153.9		[54]
Xanthate-modified magnetic chitosan	5		34.5		[68]
Thiourea-modified magnetic chitosan microspheres	5		66.7		[69]
α-ketoglutaric acid modified chitosan–coated magnetic nanoparticles Cu(II)	6		96.2		[70]
chitosan-coated sand	3		8.4		[71]
Alginate/phosphorylated chitin blend film	5		11.70		[72]
Chitosan/PVA (polyvinyl alcohol)	6		47.9		[73]
Chitosan coated PVC (polyvinyl chloride)	4		87.9		[74]
Chitosan	5		16.8		[75]
Chitosan	4.5		71.2		[76]
Non-cross linked chitosan	5		80		[77]
Chitosan acetate crown ether (CCTS–1)	5.6		23		[78]
Chitosan immobilized bentonite (CHB)	4		20		[79]
Chitosan-magnetite nanocomposites	5		35.5		[80]
Succinic anhydride-modified mercerized nanocellulose	5			105.3	[81]
Magnetic glycine-peptide	5			455	[55]
Gly sorbent	5	80.3	57.6	23.5	This work
HGly sorbent	5	186.5	110.3	46.4	This work

**Table 5:** Sorbent recycling–Sorption (S) and desorption (D) efficiencies (%) over five cycles (conditions for sorption/desorption with concentrating effect: volume of desorption corresponds to 1/10 volume of sorption).

Metal	Sorbent	Cycle #1		Cycle #2		Cycle #3		Cycle #4		Cycle #5	
		S	D	S	D	S	D	S	D	S	D
U(VI)	Gly	97.6	91.0	96.3	97.9	96.1	98.2	92.5	98.5	88.9	97.7 (±3.6)
		(±1.8)	(±4.7)	(±0.1)	(±0.9)	(±0.2)	(±0.6)	(±0.2)	(±2.6)	(±0.1)	
	HGly	97.8	95.5	97.1	98.8	94.9	97.0	92.2	95.6	92.2	95.7
		(±2.1)	(±2.6)	(±1.6)	(±2.8)	(±0.3)	(±1.0)	(±0.1)	(±1.1)	(±0.4)	(±1.8)
Cu(II)	Gly	57.7	98.7	54.8	98.0	55.4	93.6	53.8	95.0	51.1	93.2
		(±2.1)	(±0.2)	(±1.1)	(±2.2)	(±0.3)	(±0.1)	(±2.0)	(±6.4)	(±1.2)	(±3.4)
	HGly	57.5	96.8	55.3	98.8	53.6	98.4	48.7	98.0	46.1	94.4
		(±1.6)	(±0.8)	(±0.4)	(±0.4)	(±0.1)	(±0.9)	(±3.0)	(±1.0)	(±0.3)	(±2.0)
Zn(II)	Gly	70.3	87.1	69.2	86.8	67.9	87.2	66.1	90.5	63.7	93.2
		(±1.1)	(± 0.1)	(±0.9)	(± 0.8)	(±0.3)	(±2.9)	(±1.3)	(±0.1)	(±1.8)	(±3.4)
	HGly	99.9	98.8	95.2	100.2	92.9	96.8	92.2	93.3	91.0	93.1
		(±0.1)	(±1.1)	(±0.1)	(±1.8)	(±1.1)	(±1.8)	(±0.1)	(±1.0)	(±0.4)	(±0.6)

Experimental conditions: Sorption step: Contact time: 24 h; pH_0_: 5; for U(VI) *C*_0_: 26.9 mg U L^−1^; SD: 0.51 g·L^−1^ for Gly and 0.25 g·L^−1^ for HGly; for Cu(II) *C*_0_: 54 mg Cu L^−1^; SD: 1 g·L^−1^ for both Gly and HGly; for Zn(II) *C*_0_: 63.7 mg Zn L^−1^; SD: 2 g·L^−1^ for both Gly and HGly—Desorption step: contact time: 2 h, Desorption reagent: 0.2 M HCl.

## Supplementary Materials

The following are available online at www.mdpi.com/1996-1944/10/5/539/s1: Figure S1: Schematic route for the synthesis of magnetic chitosan particles and activated magnetic chitosan (with spacer arms); Figure S2: Schematic synthesis of glycine ester hydrochloride; Figure S3: Schematic route for the synthesis of Gly sorbent; Figure S4: Schematic route for the synthesis of HGly sorbent and glycine-ester magnetic-chitosan particles (intermediary product); Figure S5: Determination of pHZPC by the so-called pH drift method; Figure S6: FTIR spectra of glycine and esterified glycine; Figure S7: FTIR spectra of chitosan, magnetic chitosan, magnetic chitosan grafted with spacer arms, Gly sorbent, and HGly sorbent; Figure S8: FTIR spectra Gly sorbent before and after Zn(II), Cu(II), and U(VI) sorption and after metal desorption; Figure S9: FTIR spectra HGly sorbent before and after Zn(II), Cu(II), and U(VI) sorption and after metal; Figure S10: SEM-EDX analysis of: a) magnetic chitosan particles b) Gly c) HGly d) Gly simultaneously loaded with U(VI), Cu(II), and Zn(II) e) HGly simultaneously loaded with U(VI), Cu(II), and Zn(II) at pH 5; Figure S10: Thermogravimetric analysis (TGA and DTG) of Gly and HGly sorbents; Figure S11: pH variation during metal sorption; Table S1: Experimental frequencies for the bands observed on the FTIR spectra of glycine and glycine ester hydrochloride; Table S2: Experimental frequencies for the bands observed on the FTIR spectra of chitosan, magnetic chitosan particles, magnetic grafted chitosan (with spacer arms, via epichlorohydrin), Gly sorbent, and HGly sorbent; Table S3: Experimental frequencies for the bands observed on the FTIR spectra of Gly sorbent before and after the sorption of Zn(II), Cu(II), and U(VI) and after metal desorption; Table S4: Experimental frequencies for the bands observed on the FTIR spectra HGly before and after the sorption Zn(II), Cu(II), and U(VI) and after metal desorption. The supplementary material also includes a reminder on conventional equations used for modeling uptake kinetics (pseudo-first order rate equation and pseudo-second order rate equation) and sorption isotherms (Langmuir and Sips equation); Table S5: Effect of pH on metal speciation (main metal species and distribution percentages), at concentrations used for the study of pH effect (i.e., 100 mg Cu L^−1^; 100 mg Zn L^−1^ and 50 mg U L^−1^); Table S6: Metal speciation (main metal species and distribution percentages) at pH, for concentration ranges covering sorption isotherms.
