# Supplementary material for: Functionalization of Magnetic Chitosan Particles for the Sorption of U(VI), Cu(II) and Zn(II)—Hydrazide Derivative of Glycine-Grafted Chitosan
Source: Materials (Basel). 2017 May 16;10(5):539. doi: 10.3390/ma10050539 (PMC5459025; doi:10.3390/ma10050539)
Supplement: Supplementary file 1 [file materials-10-00539-s001.pdf]

Supplementary Materials: Functionalization of Magnetic Chitosan Particles for the Sorption of U(VI), Cu(II) and Zn(II)—Hydrazide Derivative of Glycine-Grafted Chitosan

Mohammed F. Hamza [1,2], Mohsen M. Aly [1], Adel A.-H. Abdel-Rahman [3], Samar Ramadan [3], Heba Raslan [3], Shengye Wang [2], Thierry Vincent [2] and Eric Guibal [2,*]

1. Modeling of uptake kinetics

Uptake kinetics have been modeled using both the pseudo-first order rate equation (PFORE) [1], and the pseudo-second order rate equation (PSORE) [2].

$$q(t) = q_{eq}\,[1 - e^{-k_1 t}] \quad \text{(S1)}$$

$$q(t) = \frac{q_{eq}^2\, k_2\, t}{1 + q_{eq} k_2 t} \quad \text{(S2)}$$

with q_t and q_{eq} (mg·g^{-1} or mmol·g^{-1}): sorption capacities adsorbed at t and at equilibrium, respectively. The parameters k_1 and k_2 are the rate constants of PFORE (min^{-1}) and PSORE (g·mmol^{-1} min^{-1}), respectively. The parameters of the PFORE and PSORE equations (i.e., q_{eq}, k_1 and k_2) were obtained by non-linear regression analysis using Mathematica software.

These equations have been initially designed for modeling homogeneous reaction kinetics. However, they are frequently used for describing uptake kinetics in sorption processes. Implicitly, the kinetic parameters (k_1 and k_2) are thus apparent rate coefficients that take into account the contribution of the mechanisms of resistance to diffusion (external diffusion, intraparticle diffusion).

2. Modeling of sorption isotherms

Sorption isotherms plot the sorption capacity (i.e., q_{eq}) as a function of the residual metal concentration (i.e., C_{eq}). The models most frequently used for fitting the sorption isotherms are the mechanistic equation of Langmuir and the empirical equation of Freudlich [3]. The Freundlich equation is a power-like function ($q_{eq} = k_F\, C_{eq}^{1/n}$) that does not fit experimental curves with asymptotic trends (such as those found in most solid/liquid sorption studies); contrary to the asymptotic Langmuir equation:

$$q_{eq} = \frac{q_m\, b\, C_{eq}}{1 + b\, C_{eq}} \quad \text{(S3)}$$

where q_m (mg·g^{-1}, or mmol·g^{-1}) is the maximum sorption capacity (or sorption capacity at saturation of the monolayer) and b (L·mg^{-1}, or L·mmol^{-1}) is the affinity coefficient (Langmuir constant). Frequently the Langmuir equation fails to fit the experimental points in the zone of stronger curvature of the sorption isotherms; this is especially the case for sorbents having a high affinity for target metal. The Langmuir-Freundlich equation (also called Sips equation) can be alternatively used for describing these sorption isotherms.

$$q_{eq} = \frac{q_m\, K_s C_{eq}^{1/n}}{1 + K_s\, C_{eq}^{1/n}} \quad \text{(S4)}$$

where $1/n$ is the heterogeneity factor, q_m is the total number of binding sites, and K_s (L·mg^{-1}) is the Sips affinity coefficient. The parameters of the Langmuir and Sips equations (i.e., q_m, b, K_s and n) were also calculated by non-linear regression analysis using Mathematica software.

Table S1. Experimental frequencies for the bands observed on the FTIR spectra of glycine and glycine ester hydrochloride (wavenumber, cm^{-1}).

Vibration	Ref.	Wavenumber Range (Reference)	Glycine	Glycine Ester Hydrochloride
Stretching of N–H (in NH_3^+)	[4]	3100–2600	3240–2480	3216–2568
Stretching of C–H bonds	[5]	2960	2960	2964
Stretching of C=O bonds (including C=O bond in ester)	[4]	1750–1630	1662	1741
Symmetric and asymmetric deformation of N–H (in NH_3^+)	[4,5]	1654–1511	1572	1583, 1549
Bending of C–H bonds	[5]	1465	1494	1506, 1471
Symmetric stretching of $-COO^-$ bonds	[6]	1429	1437	1454, 1411
Stretching of C–O bonds	[6]	1150–1085	1126	1135
Stretching of C–N bonds	[4]	1090–1020	1034	1051
Rocking of CH_2 groups	[4]	931	925	999, 904
Stretching of C–C bonds	[6]	896	889	854
Bending COO^- bonds	[4]	676	606	671
Wagging of COO^- bonds	[4]	584	555	591
Rocking of COO^- bonds	[4]	503	199	486

Table S2. Experimental frequencies for the bands observed on the FTIR spectra of chitosan, magnetic chitosan particles, magnetic grafted chitosan (with spacer arms, via epichlorohydrin), Gly sorbent, and HGly sorbent (wavenumber, cm^{-1}).

Vibration	Ref.	Wavenumber Range (reference)	Chitosan	Magnetic Chitosan	Magnetic Grafted Chitosan	Gly Sorbent	HGly Sorbent
Overlapping of stretching of O–H and N–H bonds	[7]	3500–3000	3650–3000	3750–3050	3750–3050	3780–3100	3750–3120
Stretching of C=O secondary amide bonds	[5,6]	1690–1630	1649	1628	1624	1626	1628
Bending of primary and secondary –OH group	[8]	1420–1330	1414, 1375, 1315	1441, 1371, 1319	1419, 1374, 1261	1456, 1375, 1319	1443, 1372
Stretching of C–O	[6,9]	1190–1130	1149, 1197	1142	1147	1149	1142
Stretching of primary C–N bonds	[9]	1090–1020	1059	1030	1061		
Antisymmetric stretching of C–O–C bonds	[10,11]	1025	1024, 993	1030	1032	1033, 1057	1030
β-D-glucose unit and rocking of CH_2	[9,12,13]	890–720	893	898	896	798, 896	897
Stretching of CH_2–Cl bonds	[9]	700–800	-	-	788	-	
Bending of free amine bond	[9]	661	657	-	-	-	-
Stretching of Fe–O bond	[13–15]	556	-	559	561	563	557

Table S3. Experimental frequencies for the bands observed on the FTIR spectra of Gly sorbent before and after the sorption of Zn(II), Cu(II), and U(VI) and after metal desorption (wavenumbers, cm^{-1}).

Vibration	Ref.	Wavenumber Range (reference)	Gly	Zn(II)-Gly	Cu(II)-Gly	U(VI)-Gly	After Metal Desorption
Overlapping of stretching of O–H and N–H bonds	[7]	3500–3000	3780–3100	3750–3234	3780–3150	3724–3080	3650–3000
Stretching of C=O bonds (secondary amide)	[5,6]	1690–1630	1626	1640	1630	1628.9	1630
Bending of O–H bonds	[8]	1420–1330	1456, 1375, 1319	-	-	-	1450, 1375, 1314
Stretching of C–N bonds (secondary amine) and stretching of C–O bonds	[6,9]	1190–1130	1149	-	-	-	1149
Antisymmetric stretching of C–O–C bonds and stretching of C–N bonds (primary amine)	[9–11]	1090–1020	1033, 1057	1011	1011	1008, 1028	1056, 1034
β-D-glucose unit and rocking of CH_2 bonds	[9,12,13]	890–720	798, 896	912	912, 786	912, 797	897
Stretching of Fe–O bonds	[13–15]	556	563	518	518	518	563
New bands related to metal sorption on NH and OH groups	[16]			740, 417	741, 422	747, 422	-

Table S4. Experimental frequencies for the bands observed on the FTIR spectra HGly before and after the sorption Zn(II), Cu(II), and U(VI) and after metal desorption (wavenumbers, cm^{-1}).

**Vibration**	**Ref.**	**Wavenumber Range (Reference)**	**HGly**	**Zn(II)-HGly**	**Cu(II)-HGly**	**U(VI)-HGly**	**After Metal Desorption**
Overlapping of stretching of O–H and N–H bonds	[7]	3500–3000	3750–3120	-	-	-	2750–3190
Stretching of C=O bonds (secondary amide)	[5,6]	1690–1630	1628	1626	1626	1622	1626
Bending of O–H bonds	[8]	1420–1330	1443, 1372	1458, 1375, 1321	1529, 1323, 1364	1527, 1327, 1325	1365, 1323
Stretching of C–N bonds (secondary amine) and stretching of C–O bonds	[6,9]	1190–1130	1142	1147	1147	1147	1151
Antisymmetric stretching of C–O–C bonds and stretching of C–N bonds (primary amine)	[9–11]	1090–1020	1030	1032, 1059	1033, 1057	1053, 1033	1055, 1032
β-D-glucose unit and rocking of CH_2 bonds	[9,12,13]	890–720	897	900	825, 897	897	896, 825
Stretching of Fe–O bonds	[13–15]	556	557	552	565	557	563
New bands related to metal sorption on NH and OH groups	[16]			417, 445	441, 428	424	

Table S5. Effect of pH on metal speciation (main metal species and distribution percentages), at concentrations used for the study of pH effect (i.e., 100 mg Cu L^{-1}, 100 mg Zn L^{-1}, and 50 mg U L^{-1})

**Metal ion**	**pH**	**Identification of main metal species and their fractions in the solution (%)**[(a)]								
Cu(I)		Cu^{2+}	$Cu(OH)^{+}$	$Cu_2(OH)^{3+}$	$Cu_2(OH)_2^{2+}$	$Cu_2(OH)_2^{2+}$	$CuCl^{+}$	$CuCl_2$		
	1	91.48	-	-	-	-	8.35	0.17		
	2	98.28	-	-	-	-	1.71	-		
	3	99.39	-	-	-	-	0.61	-		
	4	99.50	0.03	-	-	-	0.48	-		
	5	99.20	0.25	-	0.07	-	0.46	-		
	6	90.53	2.31	0.05	6.17	0.50	0.42	-		
Zn(II)		Zn^{2+}	$ZnCl_3^{-}$	$ZnCl_2$	$ZnCl^{+}$	$ZnCl_4^{2-}$	$Zn(OH)^{+}$			
	1	87.48	0.13	0.85	11.52	0.02	-			
	2	97.53	-	0.03	2.44	-	-			
	3	99.14	-	-	0.86	-	-			
	4	99.32	-	-	0.67	-	-			
	5	99.34	-	-	0.65	-	-			
	6	99.27	-	-	0.65	-	0.08			
U(VI)		UO_2^{2+}	$(UO_2)_3(OH)_5^{+}$	$(UO_2)_4(OH)_7^{+}$	$(UO_2)_2(OH)_2^{2+}$	$(UO_2)_3(OH)_4^{2+}$	$(UO_2)_2(OH)^{3+}$	$(UO_2)(OH)^{+}$	UO_2SO_4	$(UO_2)(SO_4)_2^{2-}$
	1	20.8	-	-	-	-	-	-	65.28	13.92
	2	30.31	-	-	-	-	-	0.01	66.87	2.81
	3	62.36	-	-	0.03	-	0.03	0.30	37.04	0.23
	4	73.54	0.10	-	4.79	0.05	0.47	3.75	17.25	0.04
	5	12.56	53.63	7.50	14.15	2.61	0.14	6.47	2.86	-
	6	0.30	72.56	24.18	0.80	0.35	-	1.54	0.07	-

(a): for U(VI) speciation, uranyl forms polynuclear species, the percentages represent the percentage of metal under selected from and not the molar fraction of the complexes (Note: Calculations of metal speciation using Visual MINTEQ (metal salts: $CuCl_2$, $ZnCl_2$ and UO_2SO_4) (Visual MINTEQ 3.1, Jon Petter Gustafsson, KTH University, Sweden; https://vminteq.lwr.kth.se/download/, accessed: 5/3/2017)).

Table S6. Metal speciation (main metal species and distribution percentages) at pH, for concentration ranges covering sorption isotherms.

Metal ion	Tot. Conc. (mmol·L^{-1})	Identification of main metal species and their fractions in the solution (%)[(a)]						
Cu(II)		Cu^{2+}	$Cu(OH)^{+}$	$Cu_2(OH)^{3+}$	$Cu_2(OH)_2^{2++}$	$CuCl^{+}$		
	5	98.37	0.22	0.02	0.19	1.20		
	4	98.59	0.23	0.02	0.16	1.00		
	3	98.83	0.23	0.01	0.13	0.80		
	2	99.09	0.25	-	0.09	0.57		
	1	99.37	0.26	-	0.05	0.31		
Zn(II)		Zn^{2+}	$ZnCl_2$	$ZnCl^{+}$				
	6	98.00	0.02	1.98				
	5	98.26	0.01	1.72				
	4	98.54	-	1.45				
	3	98.84	-	1.15				
	2	99.17	-	0.82				
	1	99.54	-	0.45				
U(VI)		UO_2^{2+}	$(UO_2)_3(OH)_5^{+}$	$(UO_2)_4(OH)_7^{+}$	$(UO_2)_2(OH)_2^{2+}$	$(UO_2)_3(OH)_4^{2+}$	$UO_2(OH)^{+}$	UO_2SO_4
	1	5.16	60.14	14.55	10.05	3.21	2.43	4.29
	0.8	5.85	59.89	13.46	10.59	3.15	2.80	4.09
	0.6	6.90	59.27	12.08	11.32	3.05	3.36	3.83
	0.4	8.70	57.77	10.23	12.41	2.90	4.35	3.45
	0.2	12.91	53.22	7.30	14.27	2.59	6.67	2.82
	0.1	18.94	45.79	4.76	15.85	2.18	10.01	2.22

(a): for U(VI) speciation, uranyl forms polynuclear species, the percentages represent the percentage of metal under selected from and not the molar fraction of the complexes (Note: Calculations of metal speciation using Visual MINTEQ (metal salts: $CuCl_2$, $ZnCl_2$ and UO_2SO_4) (Visual MINTEQ 3.1, Jon Petter Gustafsson, KTH University, Sweden; https://vminteq.lwr.kth.se/download/, accessed: 5/3/2017)).

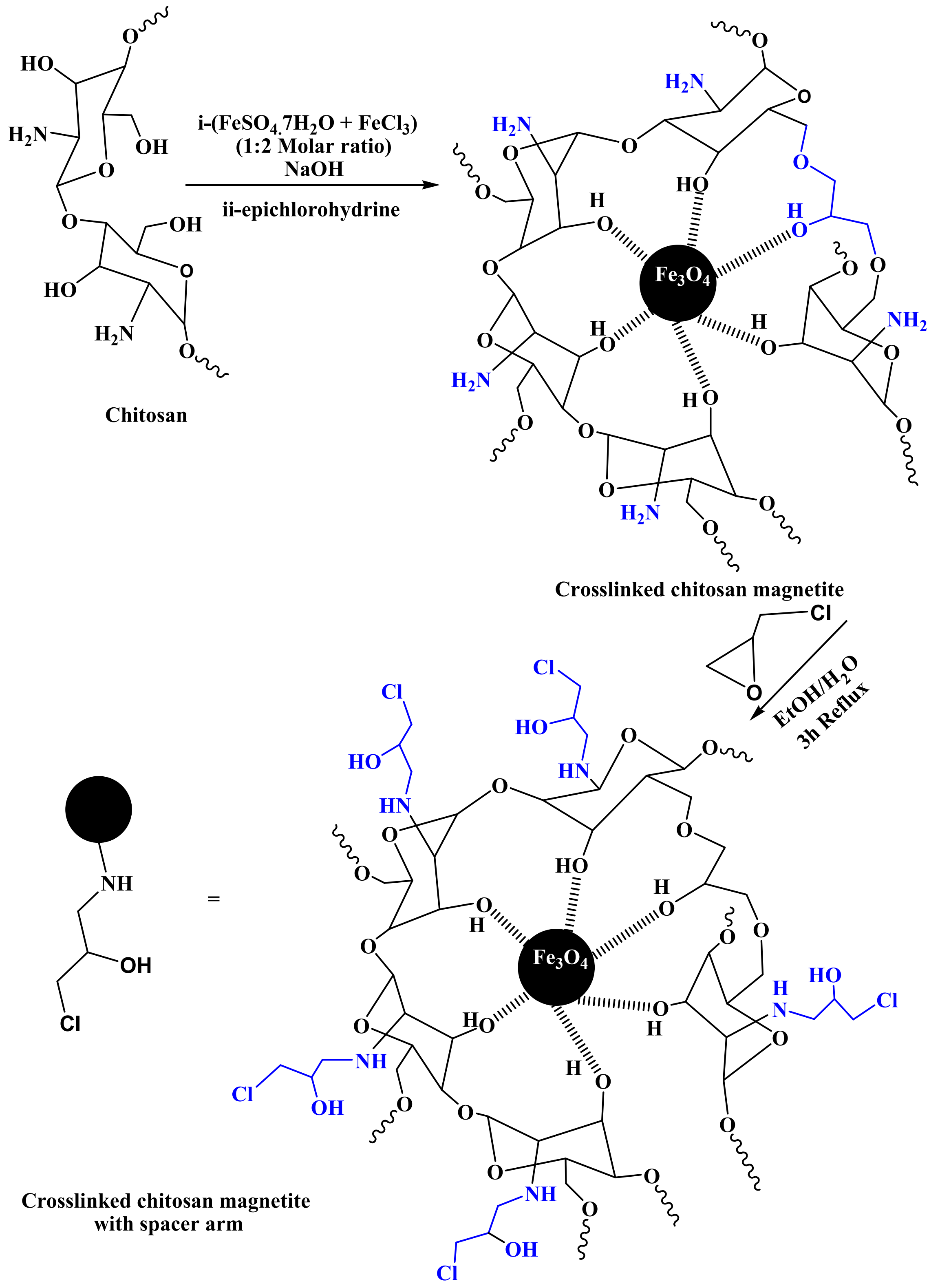

Figure S1. Schematic route for the synthesis of magnetic chitosan particles and activated magnetic chitosan (with spacer arms).

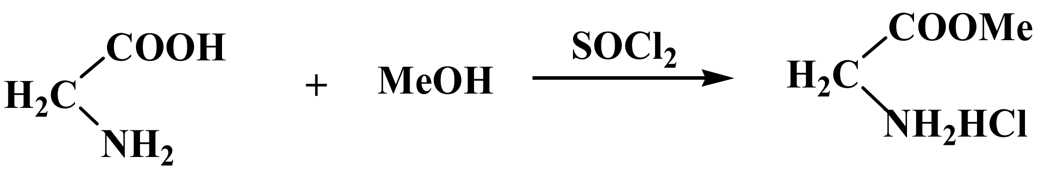

Figure S2. Schematic synthesis of glycine ester hydrochloride.

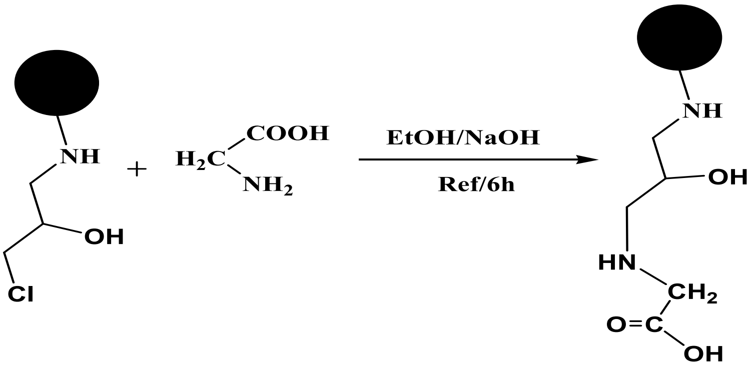

Figure S3. Schematic route for the synthesis of Gly sorbent.

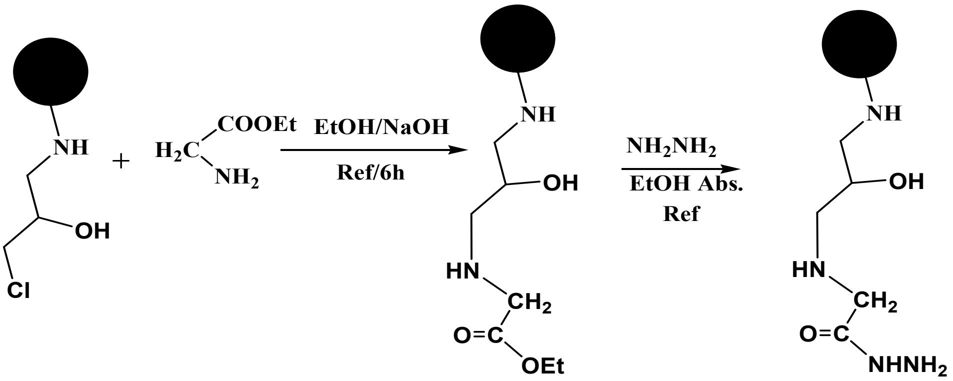

Figure S4. Schematic route for the synthesis of HGly sorbent and glycine-ester magnetic-chitosan particles (intermediary product).

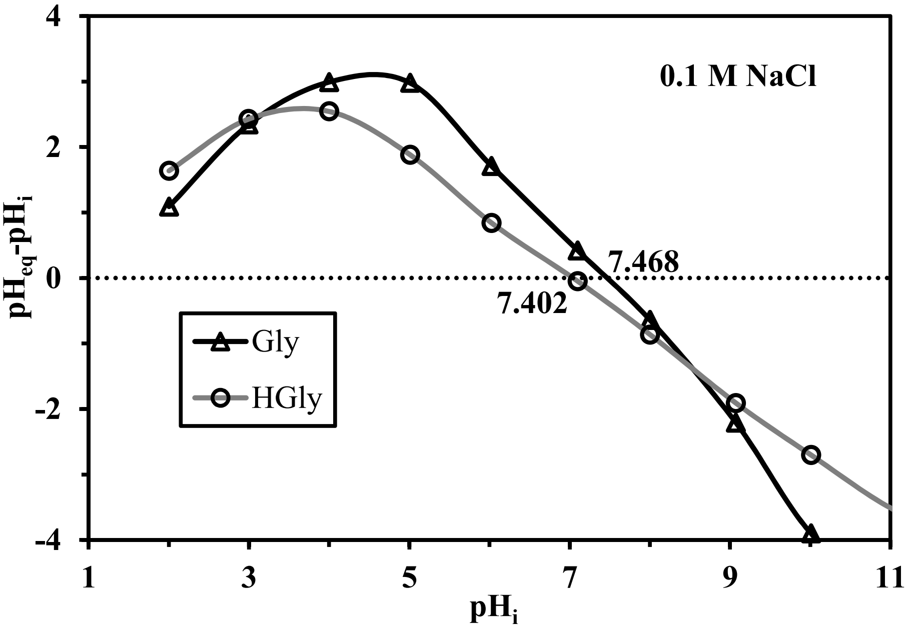

Figure S5. Determination of pH$_{ZPC}$ by the so-called pH drift method. Note: (sorbent dosage, SD: 200 mg·L^{-1}; contact time: 48 h; T: 20 °C, v: 150 rpm; C$_0$: 0.1 mol·L^{-1} NaCl).

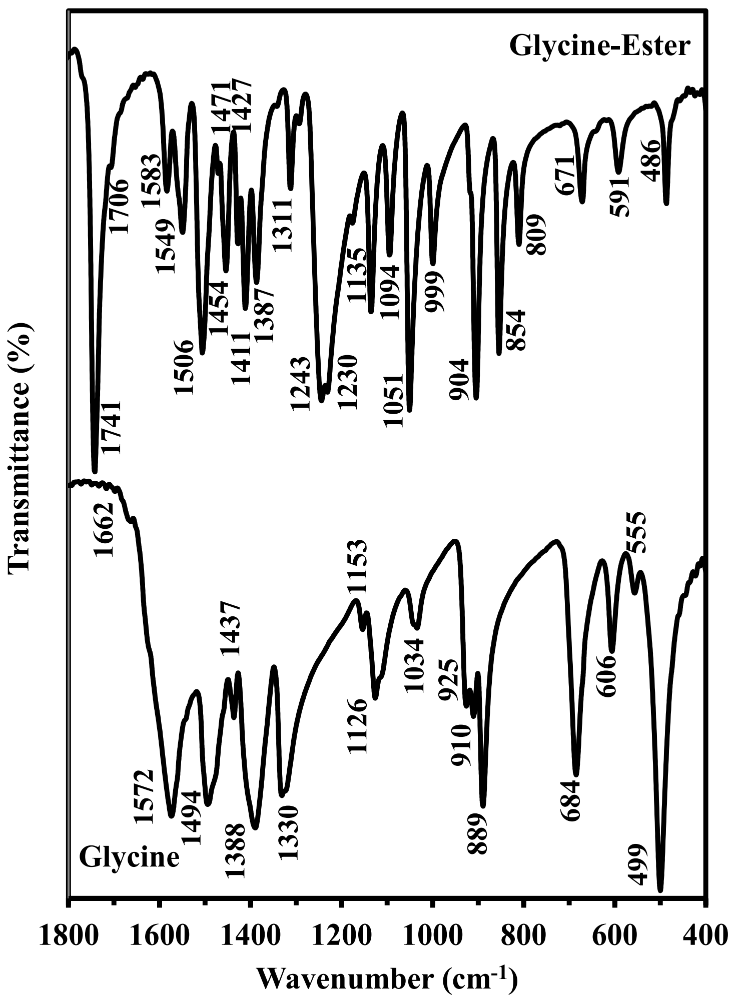

Figure S6. FTIR spectra of glycine and esterified glycine (wavenumber range: 1800–400 cm^{-1}).

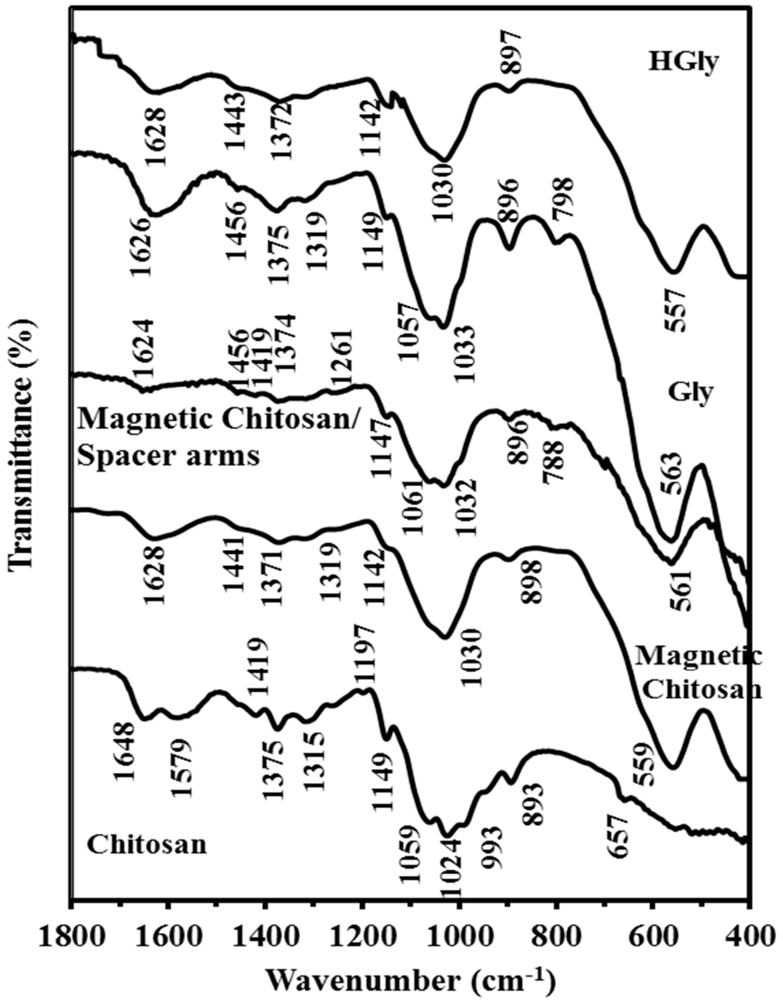

Figure S7. FTIR spectra of chitosan, magnetic chitosan, magnetic chitosan grafted with spacer arms, Gly sorbent, and HGly sorbent (wavenumber range: 1800–400 cm^{-1}).

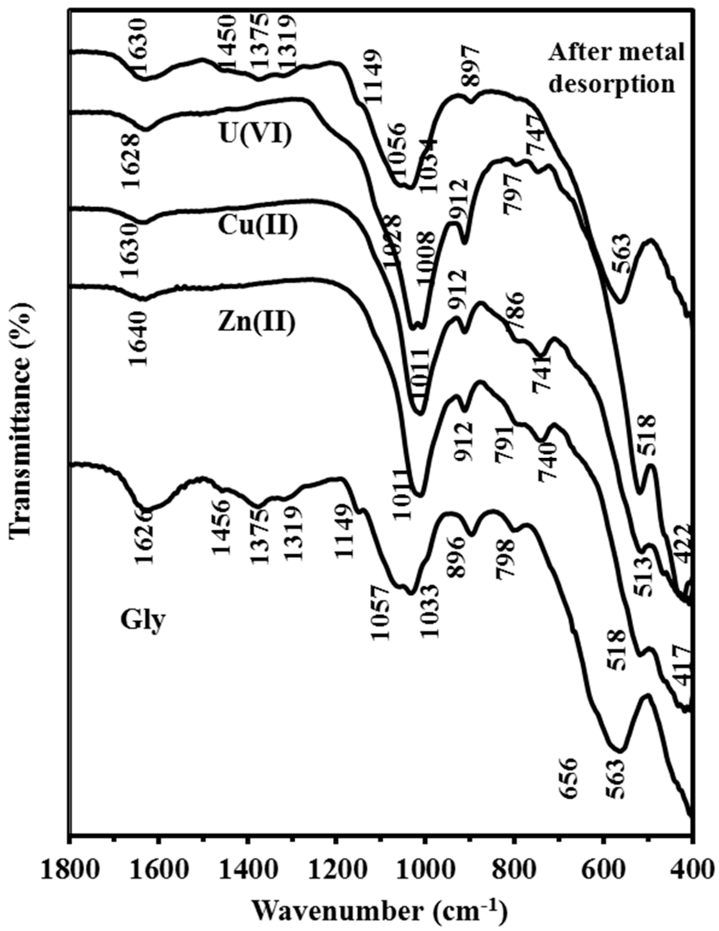

Figure S8. FTIR spectra Gly sorbent before and after Zn(II), Cu(II), and U(VI) sorption and after metal desorption (wavenumber range: 1800–400 cm^{-1}).

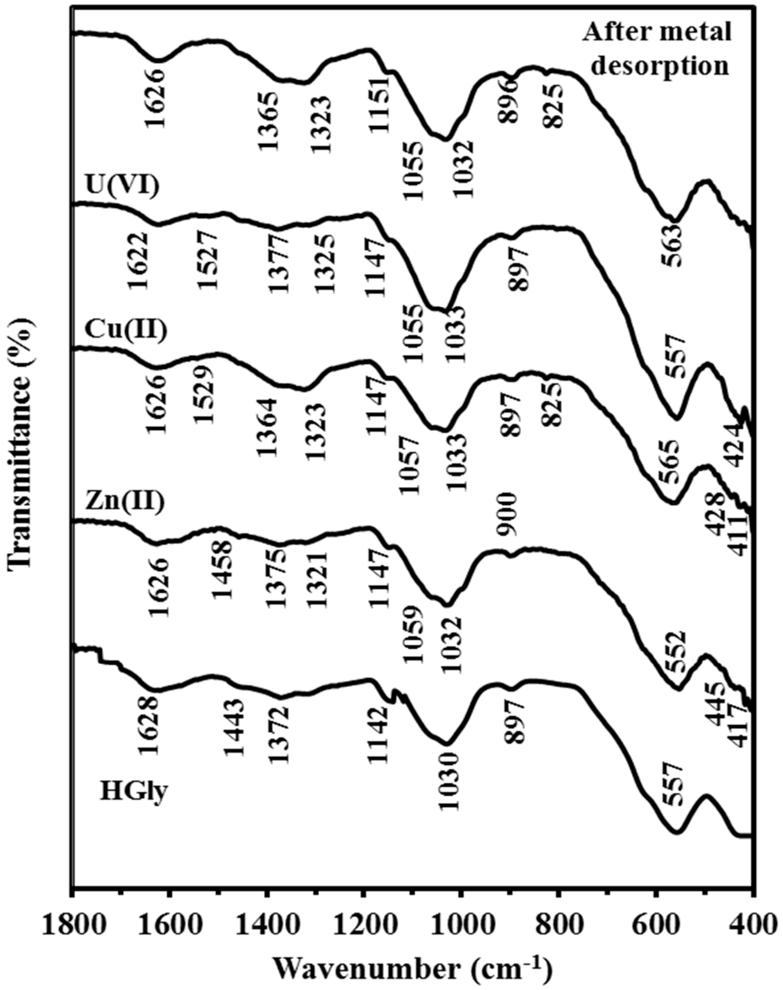

Figure S9. FTIR spectra HGly sorbent before and after Zn(II), Cu(II), and U(VI) sorption and after metal desorption (wavenumber range: 1800–400 cm^{-1}).

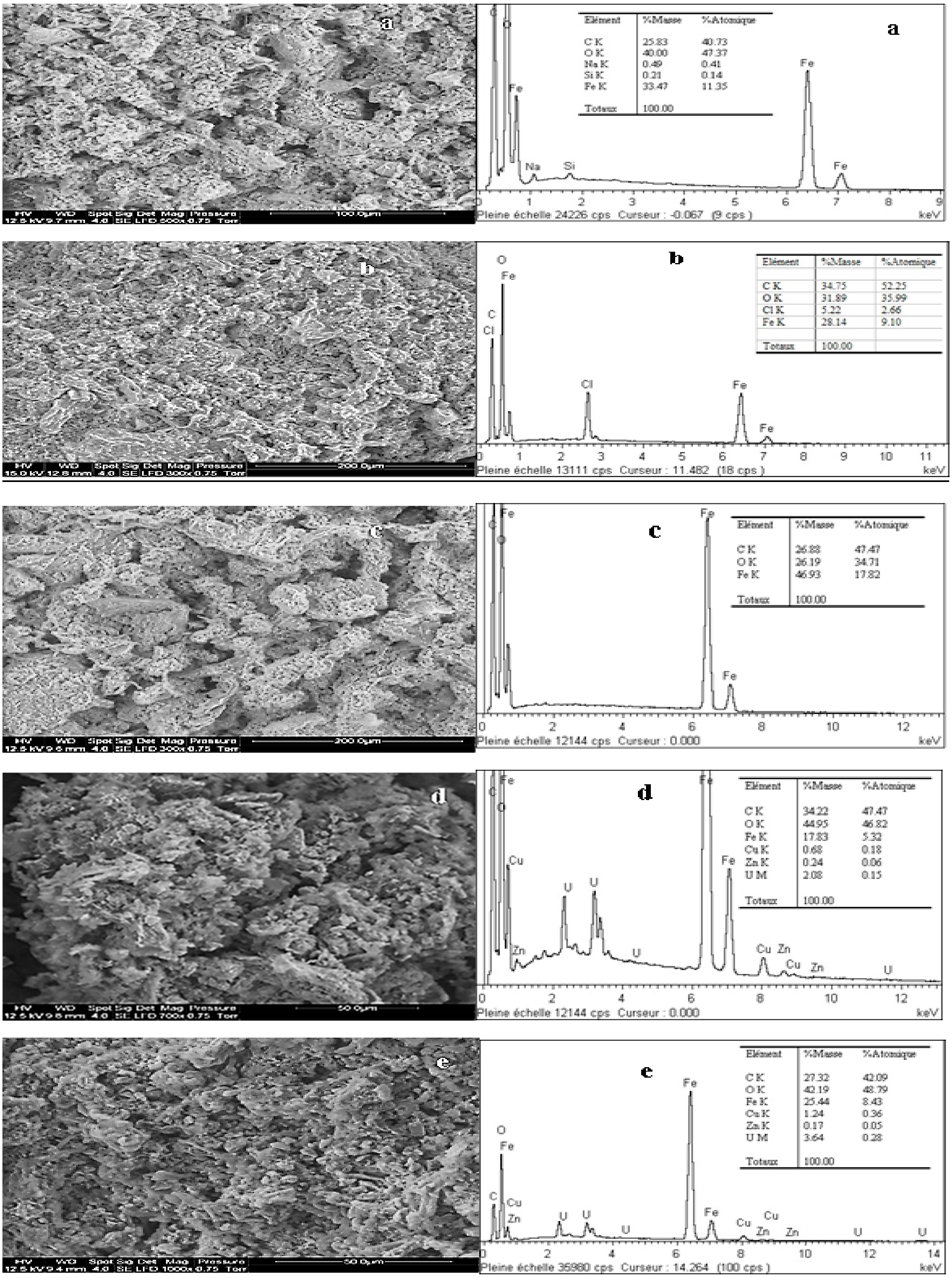

Figure S10. SEM-EDX analysis of: (**a**) magnetic chitosan particles; (**b**) Gly; (**c**) HGly; (**d**) Gly simultaneously loaded with U(VI), Cu(II), and Zn(II); (**e**) HGly simultaneously loaded with U(VI), Cu(II) and Zn(II) at pH 5.

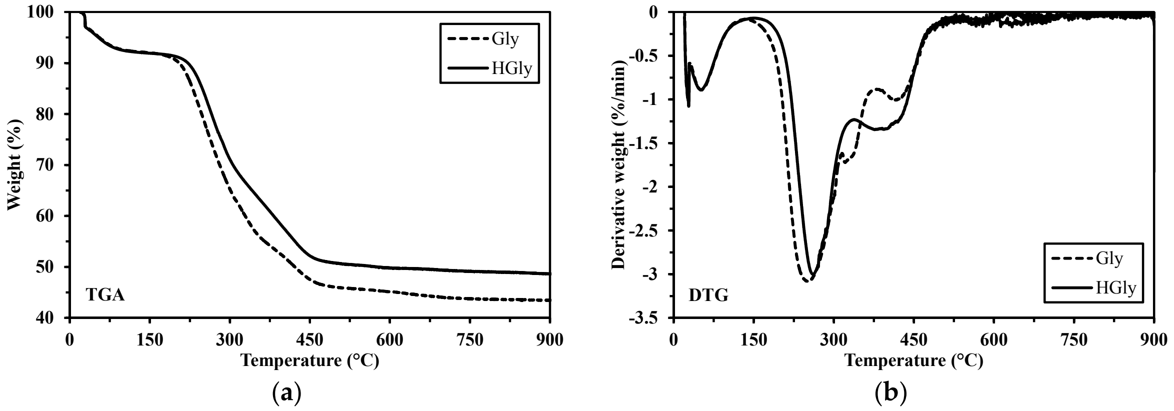

Figure S11. Thermogravimetric analysis (TGA (**a**) and DTG(**b**)) of Gly and HGly sorbents.

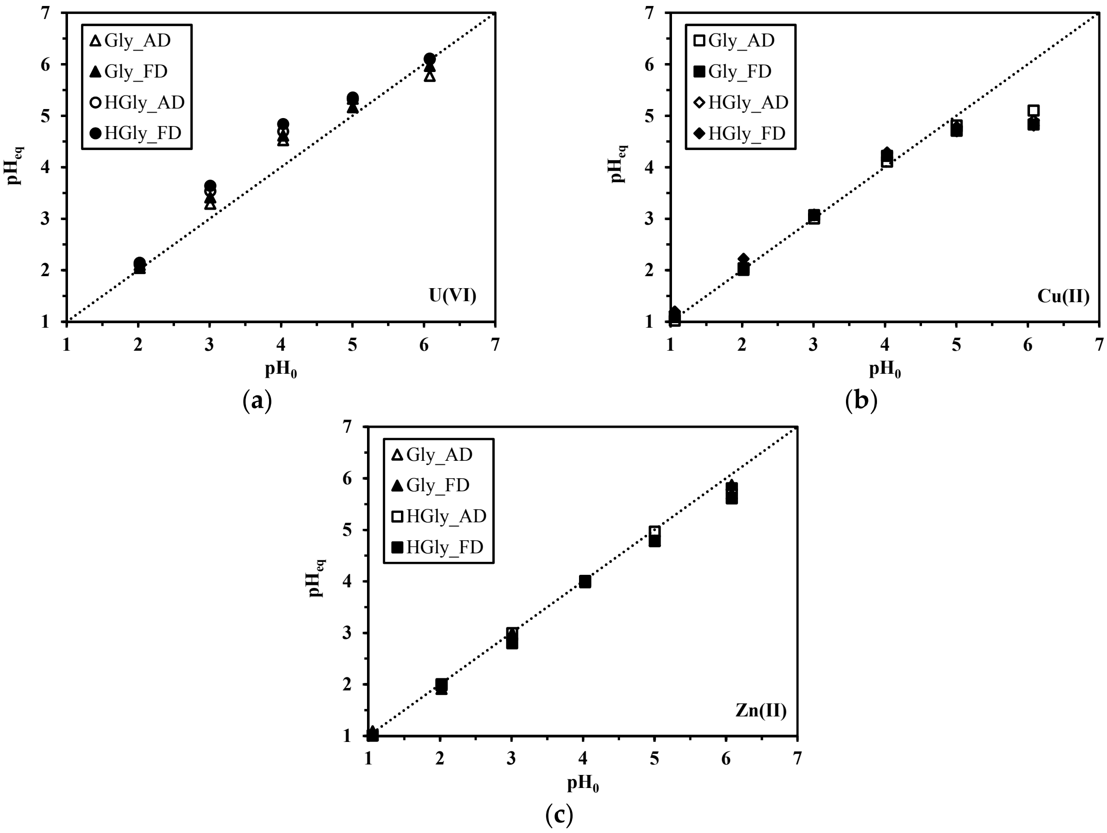

Figure S12. pH variation during metal sorption (sorbent dosage, SD: 200 mg·L^{-1}; contact time: 48 h; T: 20 °C, v: 150 rpm; (**a**): C$_0$: 50 mg U L^{-1}, (**b**): 100 mg Cu L^{-1} and (**c**): 100 mg Zn L^{-1}).

References

1. Lagergren, S., About the theory of so-called adsorption of soluble substances. *Kungliga Svenska Vetenskapsakademiens* **1898**, *24*, 1–39.
2. Ho, Y.S.; McKay, G., Pseudo-second order model for sorption processes. *Proc. Biochem.* **1999**, *34*, 451–465.
3. Tien, C., *Adsorption Calculations and Modeling*. Butterworth-Heinemann: Newton, MA, US, 1994; p. 243.
4. Hernandez-Paredes, J.; Glossman-Mitnik, D.; Esparza-Ponce, H.E.; Alvarez-Ramos, M.E.; Duarte-Moller, A. Band structure, optical properties and infrared spectrum of glycine-sodium nitrate crystal. *J. Mol. Struct.* **2008**, *875*, 295–301.

5. Xavier, T.S.; Kenny, P.T.M.; Manimaran, D.; Joe, I.H. FT-IR and Raman spectroscopic and DFT studies of anti-cancer active molecule N-{(meta-ferrocenyl) Benzoyl}—L-Alanine—Glycine ethyl ester. *Spectrochim. Acta Part A* **2015**, *145*, 523–530.
6. Mohammadi, N.; Ganesan, A.; Chantler, C.T.; Wang, F. Differentiation of ferrocene D-5d and D-5h conformers using IR spectroscopy. *J. Organomet. Chem.* **2012**, *713*, 51–59.
7. Venkatesan, G.; Pari, S. Growth of glycine ethyl ester hydrochloride and its characterizations. *Physica B-Condensed Matter* **2016**, *501*, 26–33.
8. Hu, X.-J.; Wang, J.-S.; Liu, Y.-G.; Li, X.; Zeng, G.-M.; Bao, Z.-L.; Zeng, X.-X.; Chen, A.-W.; Long, F. Adsorption of chromium (VI) by ethylenediamine-modified cross-linked magnetic chitosan resin: Isotherms, kinetics and thermodynamics. *J. Hazard. Mater.* **2011**, *185*, 306–314.
9. Coates, J. Interpretation of Infrared Spectra, A Practical Approach. In *Encyclopedia of Analytical Chemistry*, Meyers, R.A. Ed. John Wiley & Sons Ltd: Chichester, UK, 2000; pp. 10815–10837.
10. Hosoba, M.; Oshita, K.; Katarina, R.K.; Takayanagi, T.; Oshima, M.; Motomizu, S. Synthesis of novel chitosan resin possessing histidine moiety and its application to the determination of trace silver by ICP-AES coupled with triplet automated-pretreatment system. *Anal. Chim. Acta* **2009**, *639*, 51–56.
11. Oshita, K.; Takayanagi, T.; Oshima, M.; Motomizu, S. Adsorption behavior of cationic and anionic species on chitosan resins possessing amino acid moieties. *Anal. Sci.* **2007**, *23*, 1431–1434.
12. Wang, G.H.; Liu, J.S.; Wang, X.G.; Xie, Z.Y.; Deng, N.S. Adsorption of uranium (VI) from aqueous solution onto cross-linked chitosan. *J. Hazard. Mater.* **2009**, *168*, 1053–1058.
13. Xue, X.; Wang, J.; Mei, L.; Wang, Z.; Qi, K.; Yang, B. Recognition and enrichment specificity of Fe_3O_4 magnetic nanoparticles surface modified by chitosan and *Staphylococcus aureus* enterotoxins A antiserum. *Colloids Surf. B* **2013**, *103*, 107–113.
14. Namdeo, M.; Bajpai, S.K. Chitosan-magnetite nanocomposites (CMNs) as magnetic carrier particles for removal of Fe(III) from aqueous solutions. *Colloids Surf. A* **2008**, *320*, 161–168.
15. Zhang, X.; Jiao, C.; Wang, J.; Liu, Q.; Li, R.; Yang, P.; Zhang, M. Removal of uranium(VI) from aqueous solutions by magnetic Schiff base: Kinetic and thermodynamic investigation. *Chem. Eng. J.* **2012**, *198*, 412–419.
16. Kumar, P.A.; Pisipati, V. Synthesis and characterization of novel metallomesogens: La(III), Pr(III) and Nd(III) complexes of N-(2-hydroxy-4-n-alkoxy-benzaldehydeimino)-2-benzamidoethanamide. *Synth. React. Inorg. Met. Org. Chem.* **2000**, *30*, 1099–1112.

© 2017 by the authors. Submitted for possible open access publication under the terms and conditions of the Creative Commons Attribution (CC BY) license (http://creativecommons.org/licenses/by/4.0/).
